# Protective Role of Melatonin Against Postmenopausal Bone Loss *via* Enhancement of Citrate Secretion From Osteoblasts

**DOI:** 10.3389/fphar.2020.00667

**Published:** 2020-05-19

**Authors:** Wacili Da, Lin Tao, Kaicheng Wen, Zhengbo Tao, Shaojie Wang, Yue Zhu

**Affiliations:** ^1^Department of Orthopedics, First Affiliated Hospital of China Medical University, Shenyang, China; ^2^School of Pharmaceutical Engineering, Shenyang Pharmaceutical University, Shenyang, China

**Keywords:** osteoporosis, melatonin, citrate, mineralization, remodeling

## Abstract

A negative correlation exists between the severity of osteoporosis and citrate levels in bone. Our previous research found that melatonin can significantly improve bone mass in mice with osteoporosis, but the underlying mechanism involving citrate remains unknown. Herein, we demonstrated that melatonin increased bone volume and citrate levels in ovariectomized osteoporosis mice. Melatonin increased citrate and mineralized nodules in osteoblasts induced from primary mouse bone marrow mesenchymal stem cells *in vitro*. ZIP-1 knockdown and overexpression confirmed that melatonin specifically upregulated ZIP-1 to rescue citrate levels and bone mass. In general, we verified that melatonin can improve bone mass by enhancing matrix mineralization, which is highly related to increased citrate secretion from osteoblasts, and that ZIP-1 is the target of melatonin. These findings reveal another role of melatonin in regulating bone remodeling and provide a research base for its possible application in the treatment of clinical osteoporosis in the future.

## Introduction

The bone matrix is the largest citrate storage site in the whole body. Citrate is an important component of the bone matrix apatite/collagen complex and provides important biomechanical properties in bone, including stability, strength, and resistance to fracture (Hu et al., 2010; Costello et al., 2014; Guo et al., 2015). It has been reported that citrate levels in bones of osteoporosis mice and rats decreased significantly with bone loss (Chen et al., 2018). Osteoblasts are specific cells that produce citrate bound to hydroxyapatite and collagen in bone that integrate into the bone matrix, and only osteogenic differentiated bone marrow mesenchymal stem cells (BMSCs) exhibit this capacity (Franklin et al., 2014). There are two properties of citrate ensuring the process of integration: a favorable spacing between COO^−^ groups and lattice parameters in apatite, and the external-oriented CH_2_ groups improve its compatibility with the non-polar proline and alanine residues of collagen matrix (Hu et al., 2010). As the intermediate product of the mitochondrial tricarboxylic acid cycle, citrate is produced by acetyl coenzyme A and oxaloacetic acid and subsequently transformed into isocitrate by mitochondrial aconitase (m-acon). Glucose-derived mitochondrial citrate is deposited in apatite at the late stage of osteogenic differentiation of mesenchymal stem cells (MSCs) (Fu et al., 2018). Osteoblasts net citrate levels are produced *via* the upregulation of zinc transporter (ZIP-1), which results in the accumulation of intracellular Zn2+ that inhibits m-acon and eventually results in increased citrate secretion (Costello et al., 2015). Moreover, tricarboxylate transport protein (CTP) is responsible for the transport of citrate from mitochondria to the intracellular matrix to ensure the subsequent efflux, and studies have demonstrated that citrate deposition for bone apatite formation is accompanied with the upregulation of CTP during osteogenic differentiation of MSCs (Fu et al., 2018).It is clear that citrate metabolism is dysregulated in osteoporosis patients given that the content of citrate involved in bone reconstruction is significantly reduced and bone resorption is much greater than bone formation, ultimately resulting in decreased bone mass. Therefore, we seek to identify compounds that promote citrate secretion in osteoblasts that may be useful in the treatment of osteoporosis.

At present, increasing evidence shows that melatonin is closely related to the homeostasis of bone metabolism. *In vivo* studies have also shown that melatonin can restore bone loss in estrogen-deficient mice and rats and postmenopausal females (Kotlarczyk et al., 2012; Xu et al., 2018). It mainly depends on the following mechanisms based on the G-protein-coupled receptor (MT2): enhancing osteoblast activity, inhibiting osteoclast differentiation and scavenging oxygen free radicals. We also found that melatonin promotes osteoblast proliferation in a concentration-dependent manner (Liu et al., 2011; Liu et al., 2012; Xiong et al., 2015; Tao and Zhu, 2018). Notably, melatonin significantly induces osteoblast differentiation and matrix mineralization, but the underlying mechanism remains unclear (Roth et al., 1999; Park et al., 2011; Maria et al., 2018). Therefore, based on previous studies, this study intends to further elucidate the role of citrate secretion in the treatment of osteoporosis with melatonin and provide new ideas for the subsequent enhancement of the pharmacological effects of melatonin.

## Materials and Methods

### Animals and Cells

All animal experiments were approved by the Animal Care and Use Committee of the First Affiliated Hospital of China Medical University. Ten-week-old female SPF C57BL6/J mice were purchased from the Animal Research Center of the Chinese Academy of Sciences (Shanghai, China). At 12 weeks of age, mice were randomized into 3 groups (n=6 per group): sham, ovariectomized (OVX), and OVX + MT (melatonin, 60 mg/kg per day) groups. Mice in the sham group underwent a sham operation, whereas the other two groups underwent bilateral ovariectomy after being anaesthetized using a small animal anesthesia machine (Matrx, VMR, USA, the oxygen flow rate was 0.5 L/min, and the concentration of anesthetic drugs was 1%–3%). The OVX+MT group received an intraperitoneal injection of melatonin (Sigma, St. Louis, USA) at the indicated concentrations between 4:00 pm and 6:00 pm after 72 h of operation, and the sham and OVX groups received the same dose of normal saline as a control group. Animals were housed in an environment with constant temperature (23 ± 3°C) and humidity (45 ± 50%) with a 12-h/12-h light-dark cycle. Mice were given *ad libitum* access to food and water. All mice were sacrificed, and sera, femurs, and tibias were collected from mice after 8 weeks of feeding. Notably, there was no significant difference in serum melatonin concentration at the time of final execution (end of the 8 weeks) between the sham group and OVX group, while the injection of melatonin significantly increased the serum concentration compared with OVX group.

Primary BMSCs were obtained from the mouse femur under sterile conditions. A complete cell medium [α-MEM (HyClone, Logan, UT, USA), 10% fetal bovine serum (FBS, HyClone, UT, USA), and 1% penicillin/streptomycin (HyClone, UT, USA)] was used to resuspend the cells, and cells were repeatedly mixed and evenly distributed into a single cell suspension. Cells were grown at a constant temperature in an incubator (Thermo Fisher Scientific, MA, USA) at 37°C with 5% CO_2_ and saturated humidity. Cell adhesion was observed under an inverted microscope after 24–48 h of culture. Non-adherent cells were discarded, and the culture medium was changed every 2–3 d. Second-generation cells were employed to subsequent experiments. BMSCs were induced to differentiate into osteoblasts by conventional osteogenic induction medium including 10 nM dexamethasone, 50 μg/ml ascorbic acid and 10 mM β-glycerophosphate (all from Sigma, St. Louis, USA). The following experimental groups are employed: sham group; OVX group; OVX + MT (1 µM) group; OVX + MT (10 µM) group with 8 ml of culture medium (containing osteogenic medium+5 uM Zn+50 uM aspartate) changed every 2 d.

### Detection of Bone Mass

Micro computed tomography (micro CT, SkyScan1276, Bruker, Germany) was used to evaluate the microstructure of the trabeculae at the epiphysis and metaphysis of the femur with bone morphology parameters, such as bone mineral density (BMD), bone volume fraction (bone volume/tissue volume, BV/TV), trabecular number (Tb.N), trabecular separation (Tb.Sp), and trabecular thickness (Tb.Th).

### Detection of Citrate Levels

The femoral head was pulverized, and 50 mg of tissue was obtained from each group. The tissue was thoroughly ground in liquid nitrogen and homogenized buffer and then centrifuged at 13,000 rpm for 15 min at 4°C. The supernatant was transferred to a 1.5-ml EP tube and deproteinized with a perchloric acid/KOH protocol (Bio Vision, Cat. #K808-200). The cell culture medium was aspirated from the well and washed with phosphate-buffered saline (PBS). Then, the lysate was added, and the well contents were scraped to a 1.5-ml EP tube and further deproteinized. We then detected citrate levels in samples following the instructions in the Colorimetric/Fluorometric Assay Kit (Bio Vision, Catalog #K655-100).

### ALP Quantitative Detection

Third-generation BMSCs were inoculated into 24-well plates (Thermo Fisher Scientific, MA, USA) at 2×10^4^ cells/well, and the solution was changed once every 2 d. On the 7th day, the medium in the well was replaced by PBS, and 1% Triton X-100 was added to each well to lyse cells at 4°C for 30 min. The lysate was collected, and 30 µl of lysate was added per well to 96-well plates (Thermo Fisher Scientific, MA, USA). Cellular alkaline phosphatase activity was detected according to the instructions of the microplate alkaline phosphatase kit (Nanjing Jiancheng, China).

### Alizarin Red Staining

The mineralized nodule stained by alizarin red appears as orange was used to reflect the degree of osteoblast mineralization. Third-generation BMSC cells were inoculated in 24-well plates at 2 × 10^4^ cells/well. After 21 d of culture, the medium was removed. Cells were washed in PBS thrice, fixed with 95% ethanol for 15 min, and incubated with 0.1% alizarin red (Sigma, St. Louis, USA) for 15 min. The cells were allowed to dry naturally, and mineralized nodules were observed under an inverted microscope. Based on the principle of dissolving alizarin red using cetylpyridinium chloride, we added 100 g/L cetylpyridinium chloride and then detected the absorbance at 562 nm after shaking for 30 min.

### Western Blotting

Osteoblasts were first treated with radioimmunoprecipitation assay (RIPA) lysate including 50 mM Tris (pH 7.4), 150 mM NaCl, 1% NP-40, and 0.1% sodium dodecyl sulfate (SDS). Then, osteoblasts were completely lysed by ultrasound, and lysates were placed on ice for 30 min. After centrifugation at 4°C for 30 min, the supernatant was transferred to a new 1.5-ml EP tube for BCA protein quantification (Generay, Shanghai, China). Then, the protein (15 ug) was subject to gel electrophoresis and transferred to a polyvinylidene difluoride membrane. Then, the strips were sealed in packaging at room temperature for 2 h, incubated with antibodies (first antibody, 4°C, overnight, and second antibody, room temperature, 2 h) and finally exposed for observation. The following antibodies were used CTP (mitochondrial citrate transporter, Anti-SLC25a1, NBP1-31851, Novus Biologicals, USA, dilution ratio 1:200) and ZIP-1 (zinc transporter, Anti-SLC39a1, ab105416, Abcam, USA, dilution ratio 2 ug/ml).

### Immunofluorescence

The medium was aspirated, and the cells were fixed with 4% paraformaldehyde at room temperature for 15 min. Then, cells were washed thrice with PBS, and 0.1% Triton X-100 was added to permeabilize the cell membrane at room temperature for 5 min. The cells were incubated at room temperature for 60 min with immunofluorescent blocking solution and incubated with antibodies, including the primary antibody Anti-SLC39a1 (ab105416, Abcam, USA, dilution ratio 20 ug/ml) at 4 °C overnight and secondary antibody (1:500) at room temperature for 2 h. After washing in PBS and DAPI staining for 5 min, images were collected immediately using an inverted microscope (Olympus, Japan).

### Small RNA Interference and Overexpression

Cell transfections were performed using Lipofectamine^®^ 2000 according to the manufacturer’s instructions (Invitrogen, Carlsbad, CA, USA). Osteoblasts were transfected with ZIP-1 siRNA (UUCACUUCCAGCCCUACUGTT; GeneChem, Shanghai, China) and control siRNA. ZIP-1 was cloned into GV230 plasmids (GeneChem, Shanghai, China) to overexpress ZIP-1 in osteoblasts. The full-length ZIP-1 gene was amplified *via* polymerase chain reaction (PCR), and the following primers (product size 177bp) were used for PCR: forward, 5′-TTGGCTACATGTCTTCTGGACCTG-3′ and reverse, 5′-TGGACTGGTCTGTTCCTTGTAAGC-3′. The recombinant GV230-ZIP-1 plasmid was confirmed by endonuclease digestion and DNA sequencing (GeneChem, Shanghai, China) before transfection of osteoblasts with Lipofectamine 2000. The cells were transfected for 48 h before the follow-up experiment.

### Statistics

All data were expressed as the mean ± standard deviation (SD) and analyzed using GraphPad Prism 8 (San Diego, CA, USA) and SPSS 22.0 (Chicago, IL, USA). Protein bands (the duplication results of western blot are available in **Supplementary Figures 1** and **2**) were quantified using ImageJ (NIH, MD, USA). The Kolmogorov-Smirnov test was applied to verify the normality of data. Independent t tests and one-way ANOVA analyses were also performed to compare the means. Here, p < 0.05 indicated statistically significant.

## Results

### Melatonin Increased Bone Mass and Citrate Content in Postmenopausal Mice

**Figure 1:**
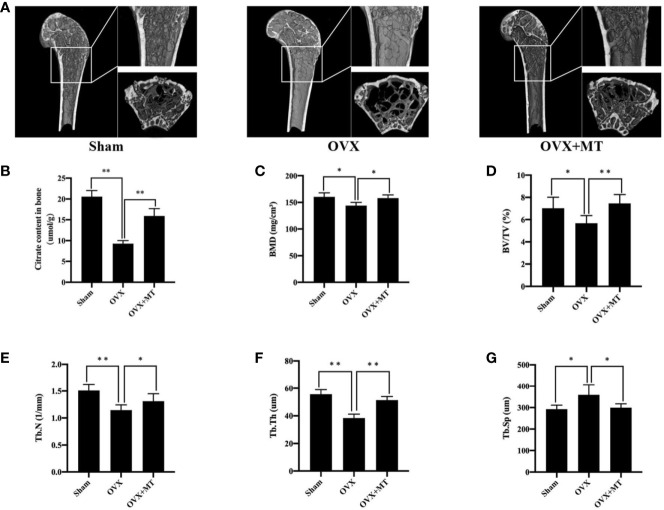
Melatonin reverses postmenopausal osteoporosis and citrate content *in vivo*. **(A)** micro-ct results of femur in different groups of mice, **(B)** citrate content in bone, **(C)** bone mineral density (BMD), **(D)** bone volume/trabecular volume (BV/TV), **(E)** trabecular number (Tb. N), **(F)** trabecular thickness (Tb. Th), and **(G)** trabecular separation (Tb. Sp). N = 6 per group. *P-value < 0.05, **P-value < 0.01. Scan parameters were set as: tomographic rotation=180°, rotation step=0.4°.

As shown in **Figure 1**, the OVX group had relatively large bone trabecular defects compared with the sham group. However, melatonin significantly increased trabecular bone and bone parameters such as BMD (P < 0.05), BV/TV (P < 0.01), Tb.N (P < 0.05), Tb.Th (P < 0.01), and Tb.Sp (P < 0.05) were significantly reduced compared with the OVX group. All of these morphological and microstructural parameters also confirmed that melatonin can protect the bone mass of osteoporosis mice. At the same time, we further found that there was a huge loss of citrate content per unit bone mass in mode animals, which could be nicely recovered with melatonin (P < 0.01) (**Figure 1B**).

### Melatonin Promoted Mineralization and Citrate Release From Osteoblasts

**Figure 2:**
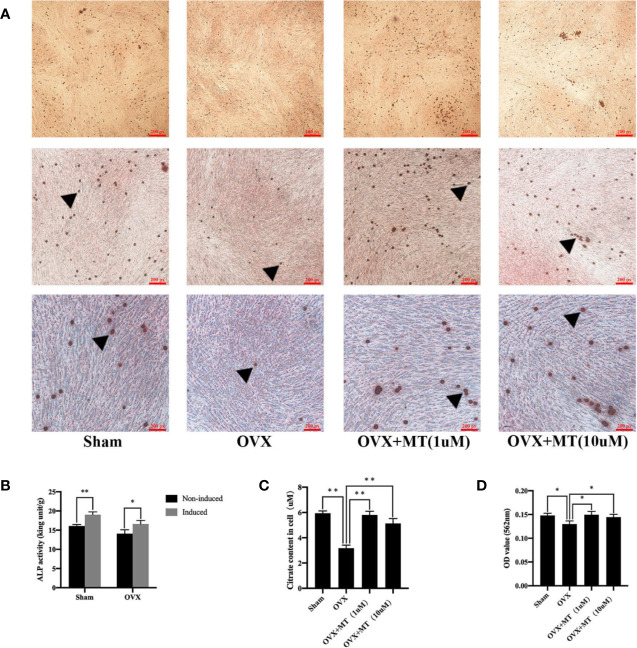
Melatonin promotes the mineralization and citrate release of osteoblasts. There were less mineralized nodules of OVX-derived mesenchymal stem cell with osteogenic induction compared with sham group, and melatonin treatment could significantly increase the nodule formation. **(A)** Alizarin red staining of mineralized nodules of osteoblasts, **(B)** detection of alkaline phosphatase (ALP) activity for osteogenesis induction, **(C)** citrate content in osteoblasts, and **(D)** absorbance of matrix mineralization at 562 nm, *P-value < 0.05, **P-value < 0.01. The triangular symbol indicates the mineralized nodules.

**Figure 2B** reveals a significant difference in ALP levels in primary BMSCs grown medium with or without an osteogenic inducer (p-value < 0.05 in the sham group and p-value < 0.01 in the OVX group). This finding indicates that BMSCs were greatly induced into osteoblasts. Exposure of osteoblasts to different concentrations of melatonin led to differences in the degree of matrix mineralization. The mineralized area stained in the drug treatment group was greater compared with the OVX group (**Figure 2A**). Quantitative absorbance test results also revealed significant differences between the drug treatment group and the OVX group (1 µM, P < 0.05; 10 µM, P < 0.05) (**Figure 2D**).

**Figure 2C** reflects the difference in citrate levels, which is remarkable between the sham and OVX groups (P-value <0.01). Moreover, both concentrations of melatonin significantly increased citrate levels compared to the levels in the OVX group. It is worth noting that citrate levels in each group are highly consistent with the degree of osteoblast matrix mineralization both *in vivo* and *in vitro*.

### Melatonin Enhanced ZIP-1 Expression

**Figure 3:**
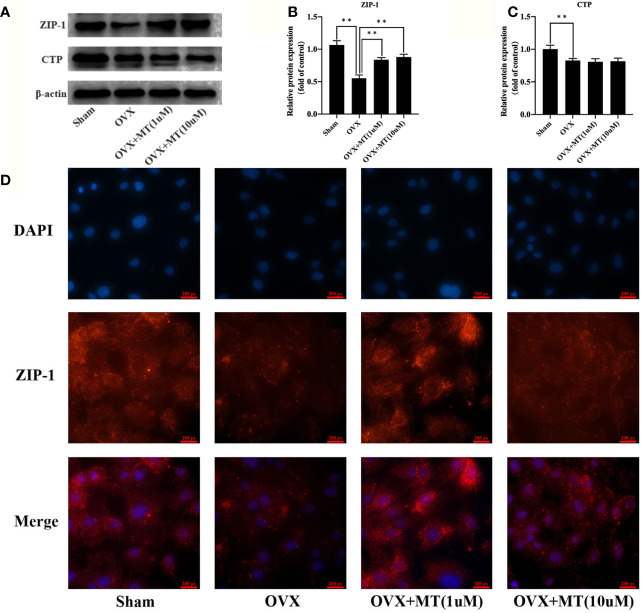
Melatonin enhances the ZIP-1 expression in osteoblasts. **(A)** western blotting results of ZIP-1 (34 kDa), CTP (34 kDa), and β-actin (42 KDa) expression in different cell groups, **(B)** relative expression value of ZIP-1, **(C)** relative expression value of CTP, and **(D)** immunofluorescence results of ZIP-1 expression in different cell groups, the expression of ZIP-1 in OVX was significantly lower than that in sham group, and the melatonin treatment could significantly promote the expression of ZIP-1.*P-value < 0.05, **P-value < 0.01.

Citrate secretion from osteoblasts is regulated by ZIP-1 and CTP. Western blotting (**Figures 3A–C**) showed that melatonin significantly upregulates ZIP-1 levels. CTP levels in the OVX group were reduced compared with the sham group, and melatonin treatment did not significantly promote CTP expression. In addition, immunofluorescence results also revealed that melatonin treatment clearly enhanced ZIP-1 expression levels in MT group compared with the OVX group (**Figure 3D**).

### Melatonin Increased Citrate Through Upregulating ZIP-1

**Figure 4:**
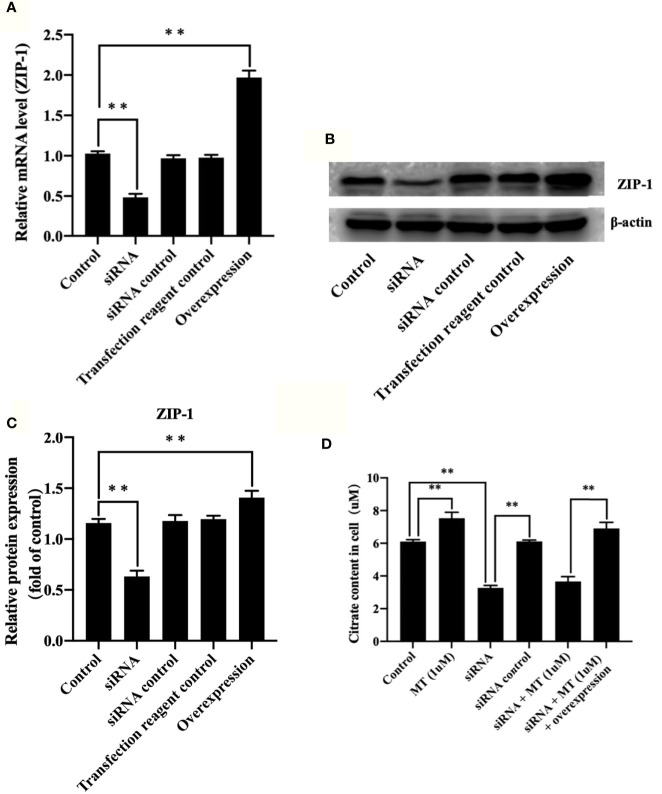
Melatonin increased citrate through up-regulating ZIP-1 in osteoblasts. **(A)** polymerase chain reaction results of ZIP-1 knockdown and overexpression, **(B)** western blotting results of ZIP-1 (34 kDa) knockdown and overexpression, **(C)** relative expression value of ZIP-1, and **(D)** citrate content in osteoblasts, *P-value < 0.05, **P-value < 0.01.

To further verify the role of ZIP-1 in melatonin-induced osteoblast differentiation, we found that compared with control (**Figures 4A–C**), ZIP-1 expression is significantly reduced after siRNA interference (P < 0.01), and ZIP-1 overexpression significantly recovers these levels (P < 0.01). As shown in **Figure 4D**, melatonin alone can remarkably improve citrate secretion compared with the control group (P < 0.01), but that effect of melatonin was not evident after siRNA interference (P < 0.01). In addition, ZIP-1 overexpression completely restored citrate levels to levels similar to that noted in melatonin alone group, indicating the critical role of ZIP-1 in regulating citrate secretion.

## Discussion

The most fundamental pathogenetic feature of osteoporosis is the imbalance between bone formation (osteoblasts) and bone resorption (osteoclasts) (Alford et al., 2015; Han et al., 2018; Hu et al., 2018). Although it has long been known that citrate accumulates in bone and osteoporosis is consistently accompanied by a significant reduction in citrate levels both *in vitro* and *in vivo*, its role in the development, maintenance, and pathology of osteoporosis remains unknown (Dickens, 1941; Chen et al., 2018). Therefore, we established a postmenopausal osteoporosis model and observed that citrate levels in osteoporotic bone decreased significantly, an imbalance occurs between osteoblasts and bone absorbing cells, and transformation of mesenchymal stem cells to osteoblasts is reduced compared with adipocytes. These findings are consistent with previous studies (Zhao et al., 2011; Chen et al., 2016). On one hand, the imbalance between bone formation and resorption can reduce the production of citrate by osteoblasts. On the other hand, differentiation of MSCs into adipocytes requires more citrate, which serves as a source of cytosolic acetyl CoA for lipid biosynthesis (Watson and Lowenstein, 1970). Our findings suggest that loss of citrate in bone may contribute to osteoporosis development. In 2013, Leslie first proposed the new concept of “Osteoblast Citration” (Costello et al., 2012). Subsequently, Renty and others also found that osteoblasts are cells that specifically produce citrate for incorporation into bone, and osteogenic differentiation of MSCs will lead to the development of citrate-producing osteoblasts (Franklin et al., 2014). Moreover, osteoblasts enable citrate to bone integration in the form of mineralization (Costello et al., 2015), which further confirmed that osteoblasts are the key cells that regulate citrate levels and bone mass. It has also been proposed that inhibition of extracellular citrate uptake is a potential treatment for metabolic diseases (Iacobazzi and Infantino, 2014; Icard et al., 2018). Therefore, we desperately need to develop a drug that can effectively promote citrate secretion from osteoblasts as a new strategy to improve the bone mass of patients with osteoporosis.

In recent years, the effect of melatonin on osteoporosis has become increasingly evident. Compared with existing traditional anti-osteoporosis drugs, melatonin not only inhibits bone loss but also promotes new bone formation (Li et al., 2019). In addition, melatonin plays multidimensional roles in metabolic disorders caused by estrogen deficiency, such as protecting the liver of OVX rats against steatosis, curing sleep disorders in age-related estrogen deficiency and correcting glycemic dysregulation (Maganhin et al., 2014; Mao et al., 2014; Hermoso et al., 2016). These metabolic disorders are not conducive to bone formation. In our study, melatonin’s positive regulation of bone mass is consistent with changes in citrate levels in bone. In addition, *in vitro* experiments also revealed that melatonin promotes citrate secretion by osteoblasts, which is also consistent with the degree of matrix mineralization. Melatonin is not only responsible for osteoblast energy metabolism but also involved in bone mineralization. Moreover, the activity of mitochondrial aconitase, which catalyzes the transformation of citrate into isocitrate, is inhibited by Zn2^+^ based on increased ZIP-1 expression (Fu et al., 2018). Despite the previous publication revealed that melatonin induces Zn2+ influx *via* ZIP-1 in N2a cells to exert a protective effect in a hypoxic environment, we sought to determine the relationship among melatonin, ZIP-1, citrate, and bone remodeling for the first time (Liu et al., 2015). Surprisingly, the increase in citrate production induced by melatonin was mainly accompanied by an increase in ZIP-1 not CTP, and further knockdown and overexpression of ZIP-1 confirmed the specificity of melatonin in regulating ZIP-1, indicating that ZIP-1 is the main genetic/metabolic factor required for osteoblasts to achieve net citrate production. The results of this study now implicate melatonin in facilitating osteoblast citration except for facilitating osteoblast differentiation.

In general, we observed that melatonin promotes citrate secretion in osteoblasts and enhances matrix mineralization. *In vivo*, we also found that the effect of melatonin on the recovery of bone mass in postmenopausal mice is highly consistent with citrate levels in bone, and its mechanism of that action is primarily related to ZIP-1 expression. These results reveal the critical role of citrate in bone homeostasis and provide the research basis for the future clinical application of citrate in osteoporosis treatment. Of note, ZIP-1 is associated with zinc (Zn2 +) levels in osteoporosis; thus, whether zinc replenishment by dietary zinc supplementation would be of value in restoring the production of citrate in bone warrants further study.

## Data Availability Statement

The datasets supporting the conclusions of this article are available from the corresponding authors upon reasonable request.

## Ethics Statement

The animal study was reviewed and approved by the Animal Care and Use Committee of the First Affiliated Hospital of China Medical University.

## Author Contributions

All authors contributed to the study conception and design. Material preparation, data collection and analysis were performed by WD, KW, ZT, and LT. The first draft of the manuscript was written by WD and all authors commented on previous versions of the manuscript. YZ and SW are responsible for monitoring the progress of the entire study. All authors read and approved the final manuscript.

## Funding

This study was funded Special Funds for Guiding Local Scientific and Technological Development by the Central Government of Liao Ning Province in China (grant number: 2019416030) and Initiation Foundation of Doctoral Research of Liao Ning Province in China (grant number: 2019-BS-294).

## Conflict of Interest

The authors declare that the research was conducted in the absence of any commercial or financial relationships that could be construed as a potential conflict of interest.

## Supplementary Material

Supplementary Figure 1 The duplication of western blot **Figure 3**.

Supplementary Figure 2 The duplication of western blot in **Figure 4**.
