# Corrigendum: Protective Role of Melatonin Against Postmenopausal Bone Loss via Enhancing Citrate Secretion in Osteoblasts

**DOI:** 10.3389/fphar.2021.652249

**Published:** 2021-03-11

**Authors:** Wacili Da, Lin Tao, Kaicheng Wen, Zhengbo Tao, Shaojie Wang, Yue Zhu

**Affiliations:** ^1^ Department of Orthopedics, First Hospital of China Medical University, Shenyang, Liaoning, China; ^2^ School of Pharmaceutical Engineering, Shenyang Pharmaceutical University, Shenyang, Liaoning, China

**Keywords:** osteoporosis, melatonin, citrate, Mineralization, Remodeling

In the original article, there was a mistake in Figures 3, 4 as published. In the process of rearranging the published articles, we found a problem by accident that we mistakenly uploaded the results of western blot bands in Figures 3, 4. Those western blot bands are uneven due to the instability of hardware and improper exposure operations, which may cause unnecessary misunderstandings for readers in the future. The corrected Figures 3, 4 and **Supplementary Material** appear below.

**FIGURE 3:**
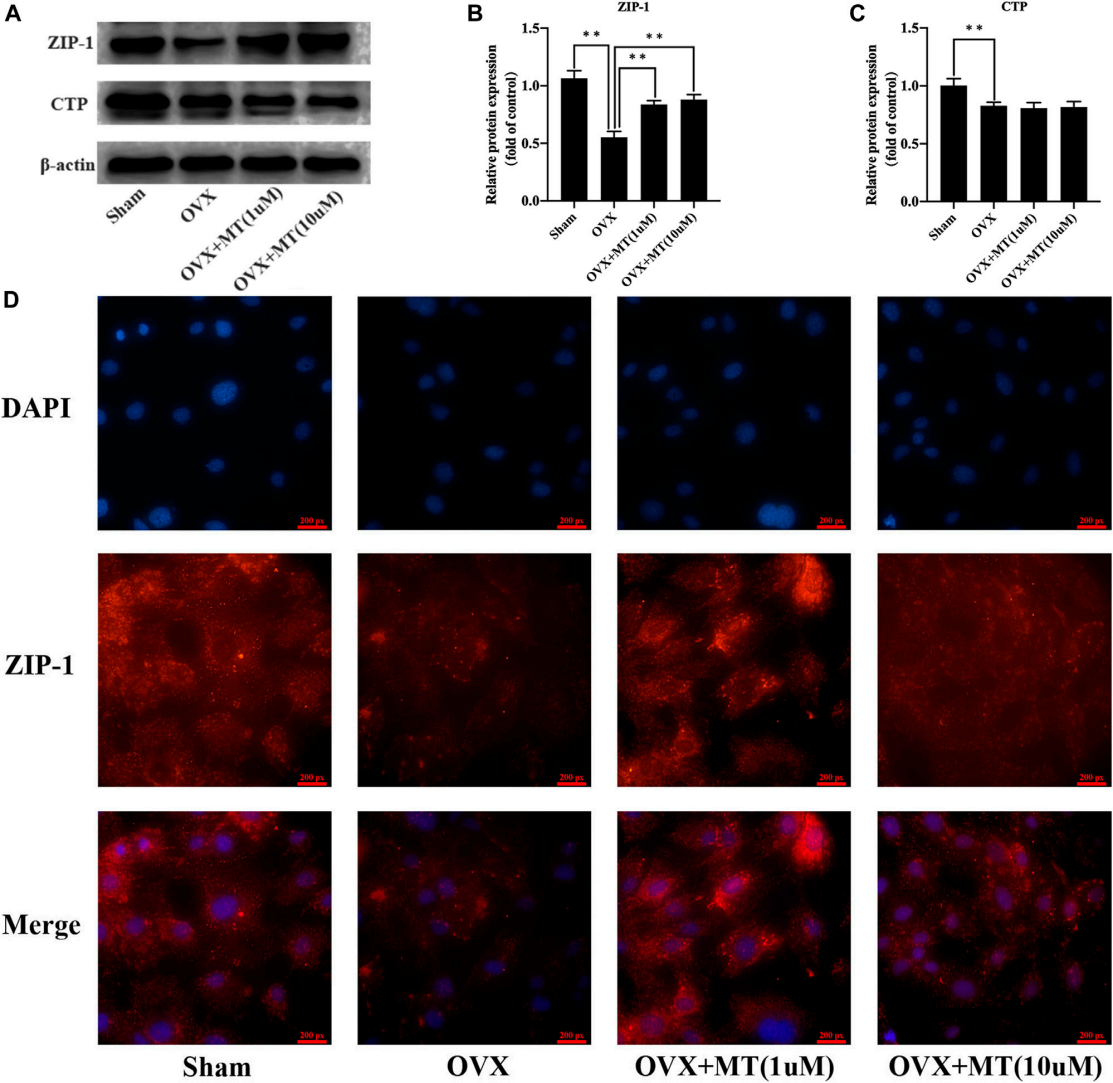
Melatonin enhances the ZIP-1 expression in osteoblasts. **(A)** western blotting results of ZIP-1 (34 kDa), CTP (34 kDa), and β-actin (42 KDa) expression in different cell groups, **(B)** relative expression value of ZIP-1, **(C)** relative expression value of CTP, and **(D)** immunofluorescence results of ZIP-1 expression in different cell groups, the expression of ZIP-1 in OVX was significantly lower than that in sham group, and the melatonin treatment could significantly promote the expression of ZIP-1. *P-value < 0.05, **P-value < 0.01.

**FIGURE 4:**
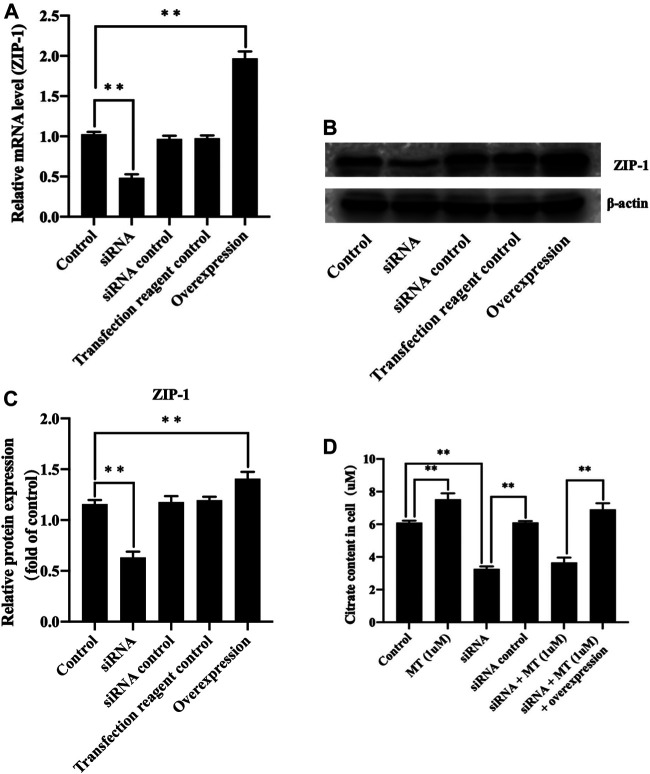
Melatonin increased citrate through up-regulating ZIP-1 in osteoblasts. **(A)** polymerase chain reaction results of ZIP-1 knockdown and overexpression, **(B)** western blotting results of ZIP-1 (34 kDa) knockdown and overexpression, **(C)** relative expression value of ZIP-1, and **(D)** citrate content in osteoblasts, *P-value < 0.05, **P-value < 0.01.

The authors apologize for this error and state that this does not change the scientific conclusions of the article in any way. The original article has been updated.

