# Supplementary figures and images for: Protective Role of Melatonin Against Postmenopausal Bone Loss via Enhancement of Citrate Secretion From Osteoblasts
Source: Front Pharmacol. 2020 May 19;11:667. doi: 10.3389/fphar.2020.00667 (PMC7248328; doi:10.3389/fphar.2020.00667)

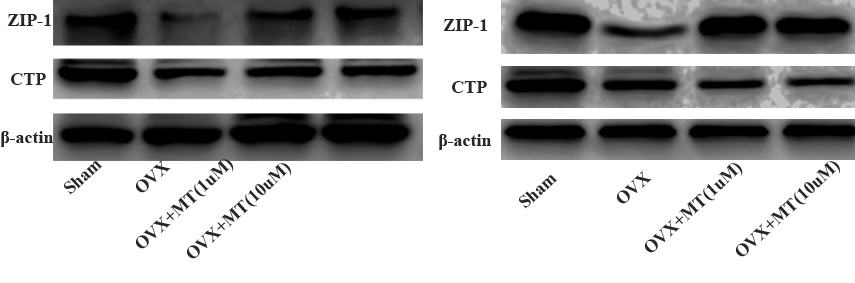

Supplement: Supplementary Figure 1 — The duplication of western blot Figure 3. [file Image_1.tif]

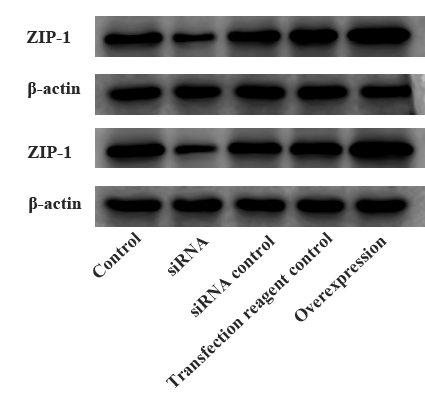

Supplement: Supplementary Figure 2 — The duplication of western blot in Figure 4. [file Image_2.tif]

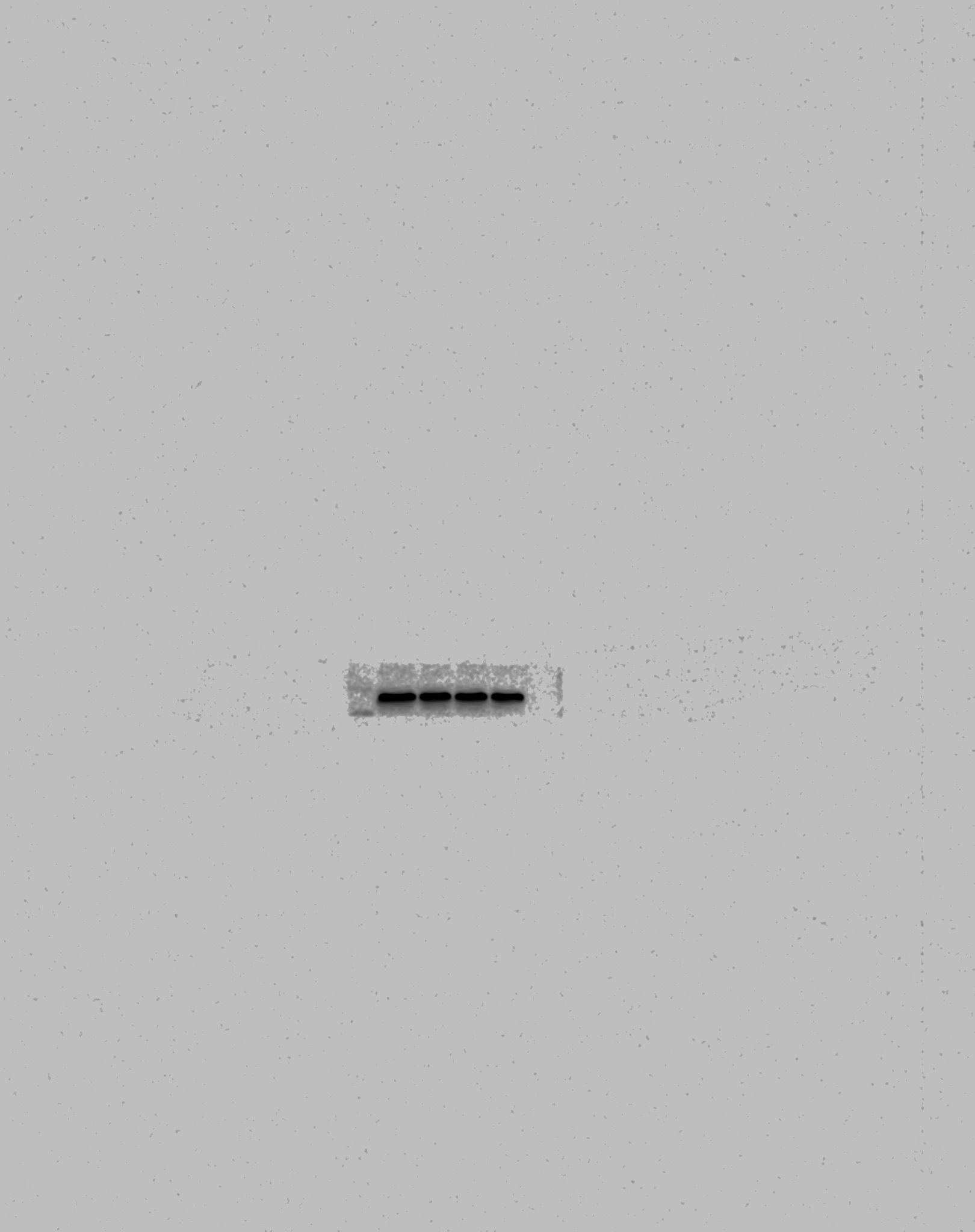

Supplement: Supplementary file 3 [file DataSheet_1.zip › figure3-actin-1.tif]

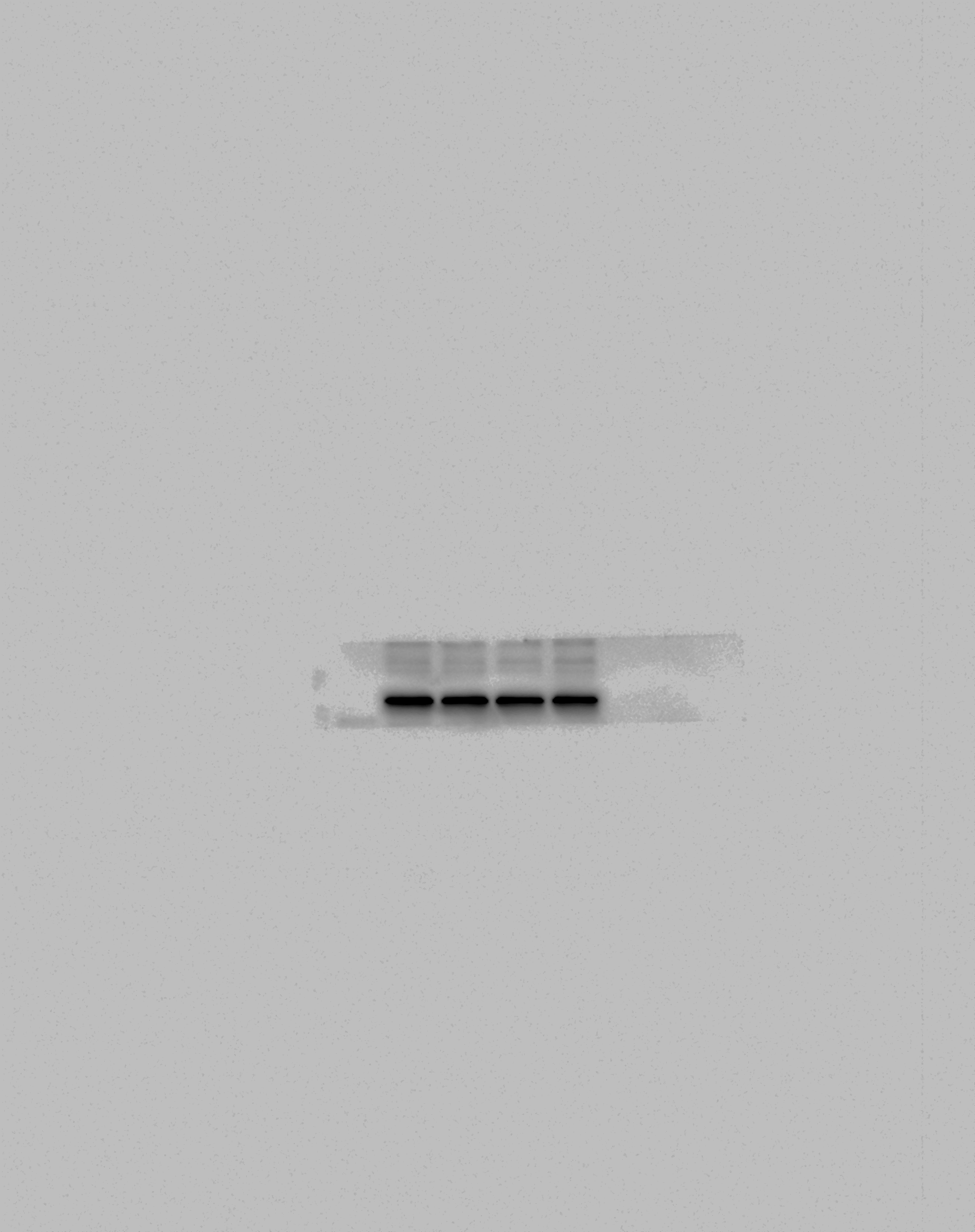

Supplement: Supplementary file 3 [file DataSheet_1.zip › figure3-actin-2.tif]

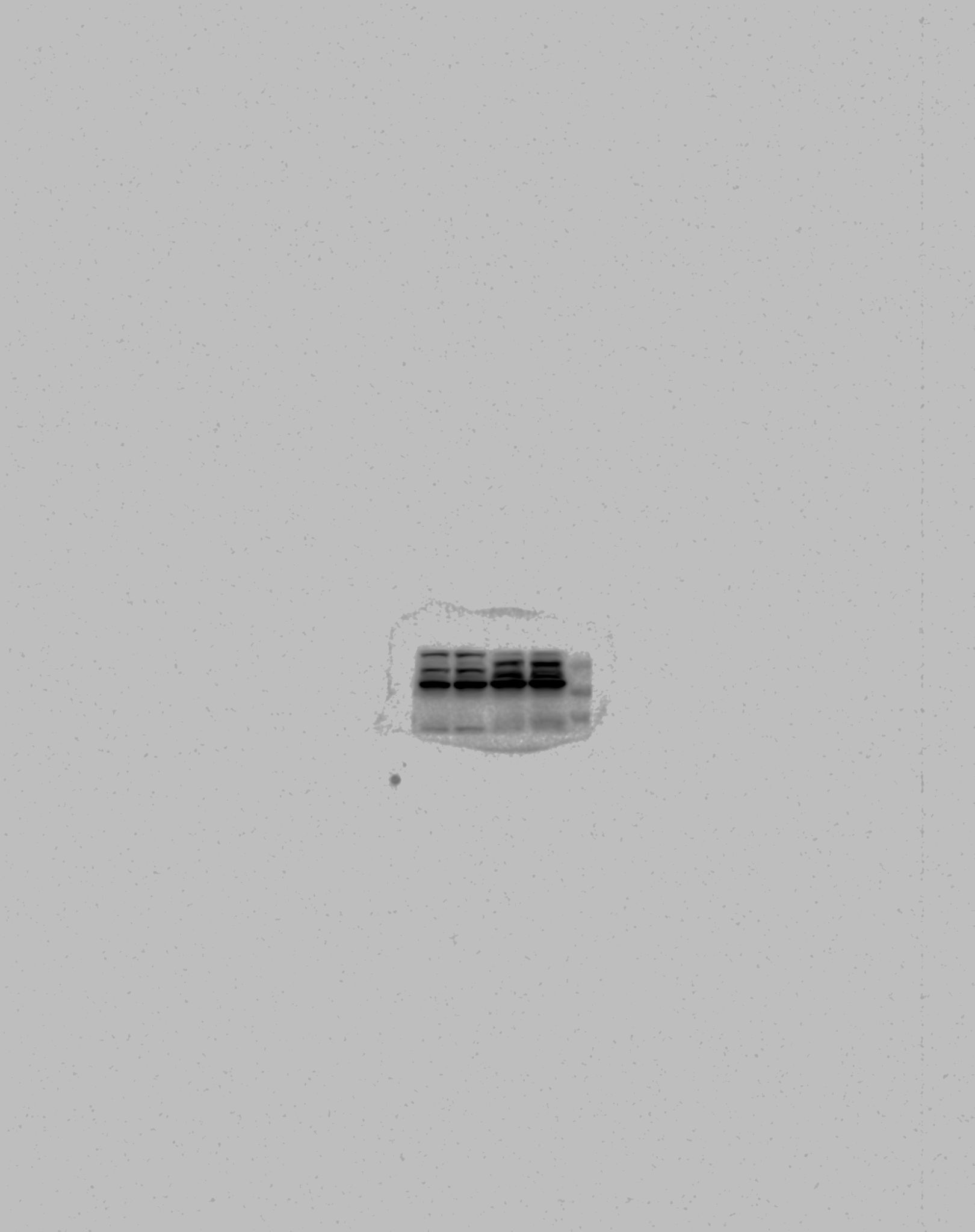

Supplement: Supplementary file 3 [file DataSheet_1.zip › figure3-actin-3.tif]

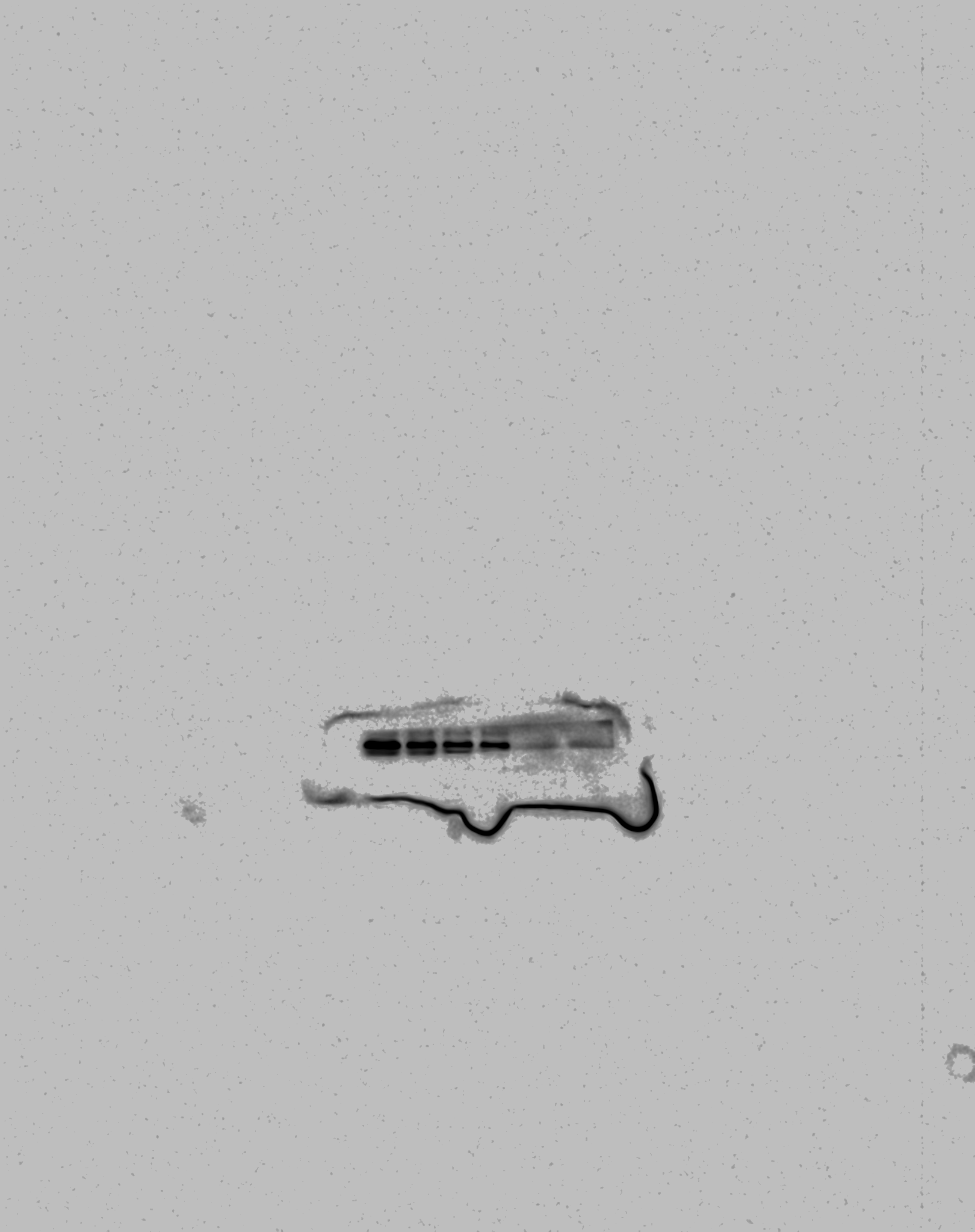

Supplement: Supplementary file 3 [file DataSheet_1.zip › figure3-CTP-1.tif]

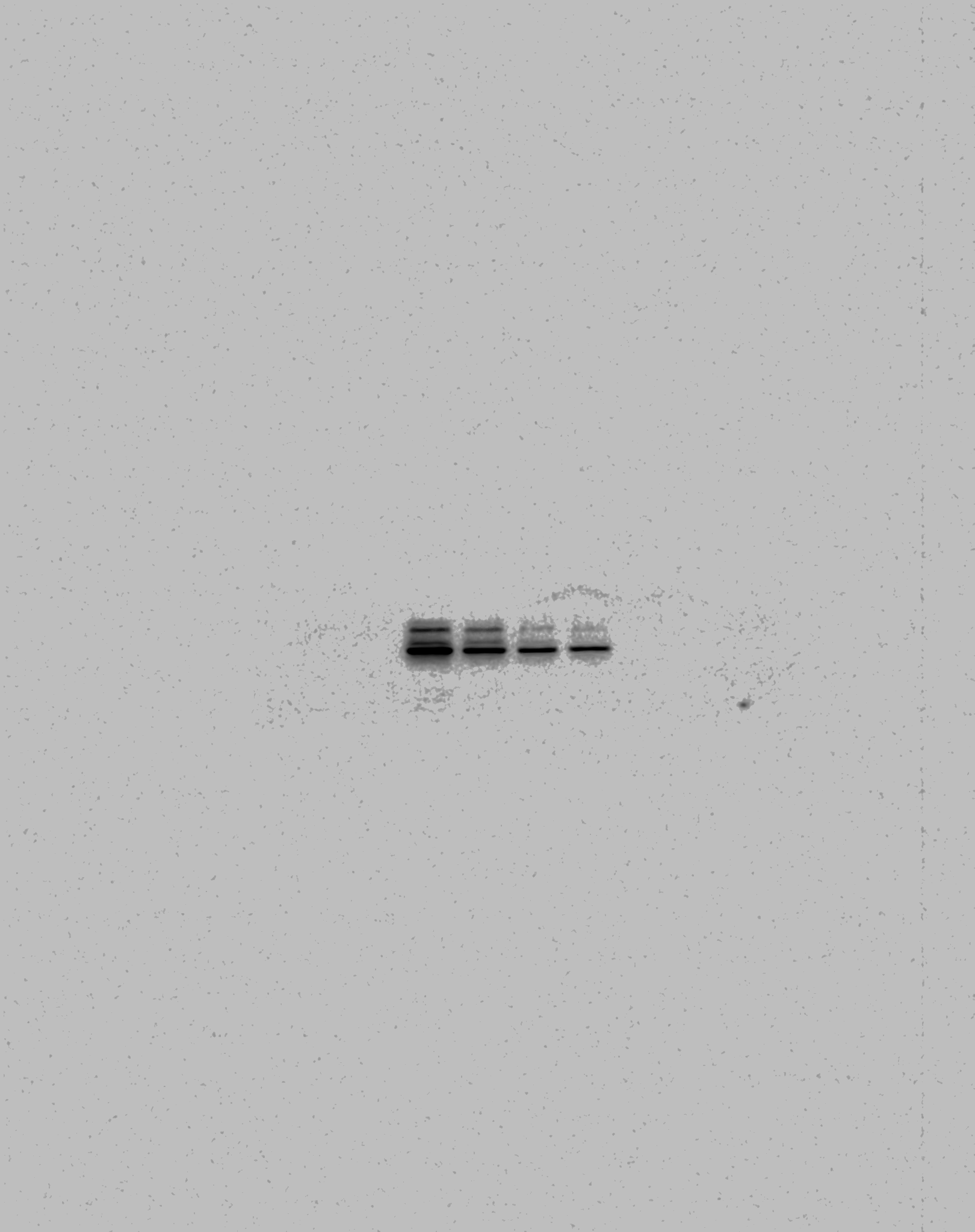

Supplement: Supplementary file 3 [file DataSheet_1.zip › figure3-CTP-2.tif]

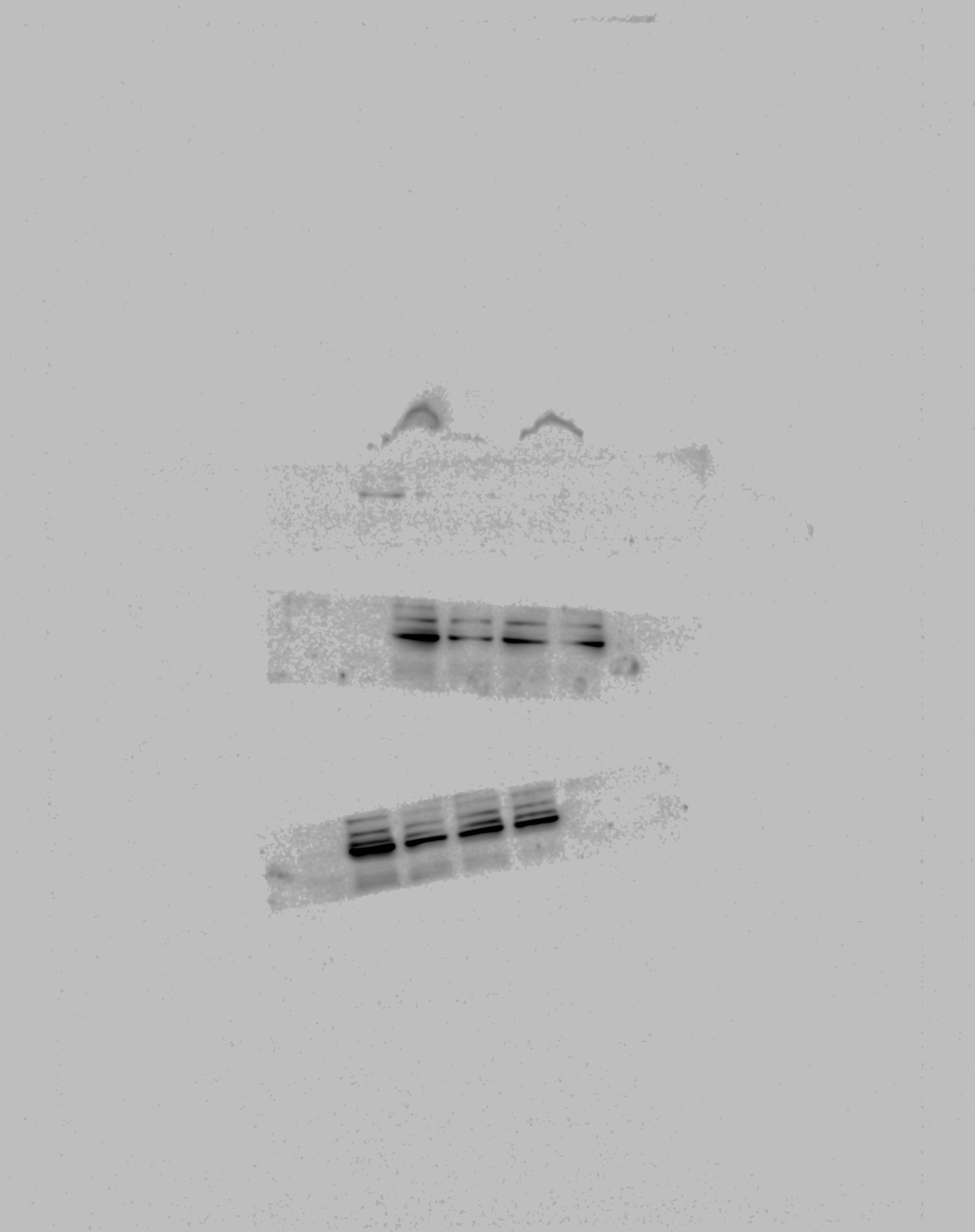

Supplement: Supplementary file 3 [file DataSheet_1.zip › figure3-CTP-3.tif]

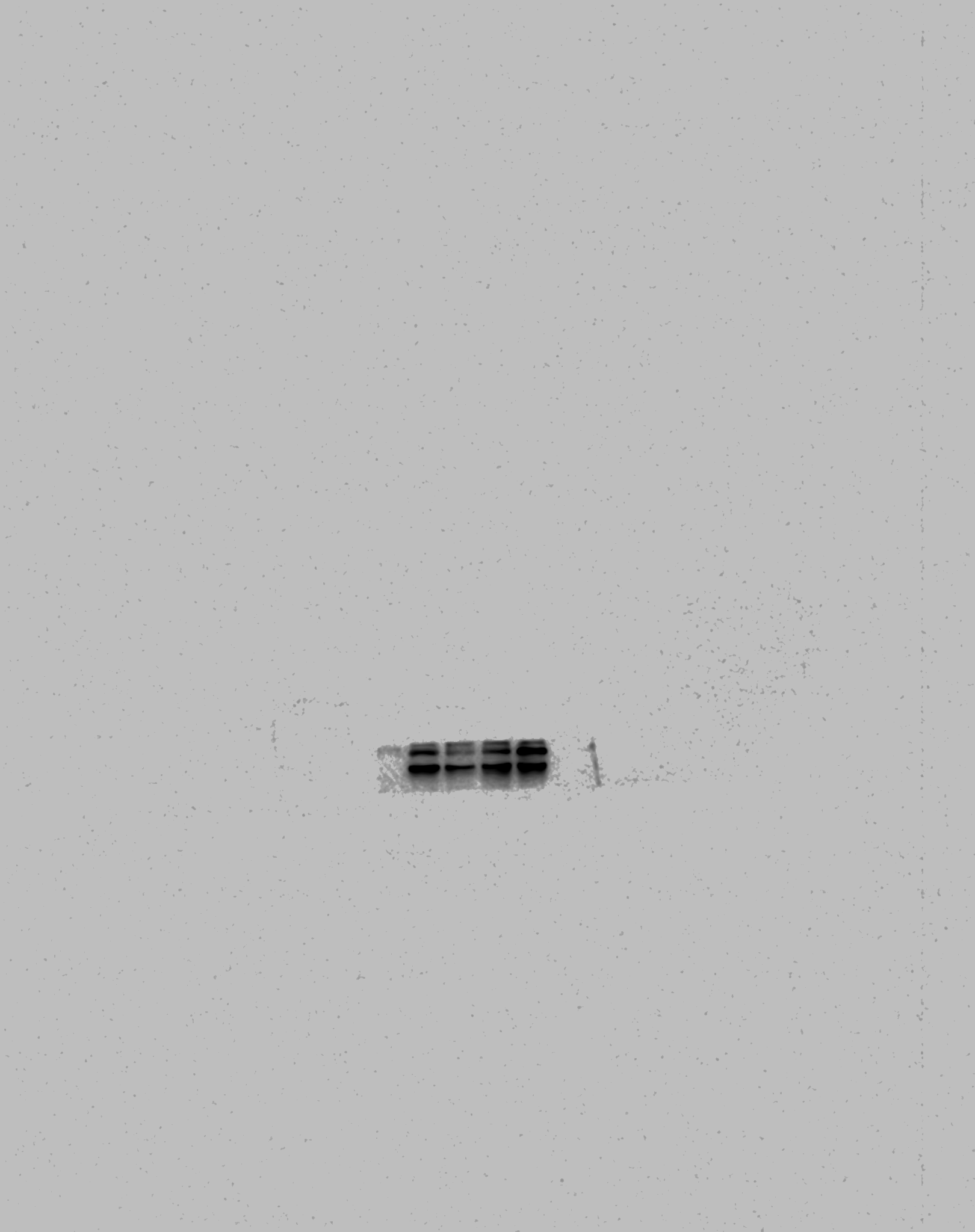

Supplement: Supplementary file 3 [file DataSheet_1.zip › figure3-ZIP1-1.tif]

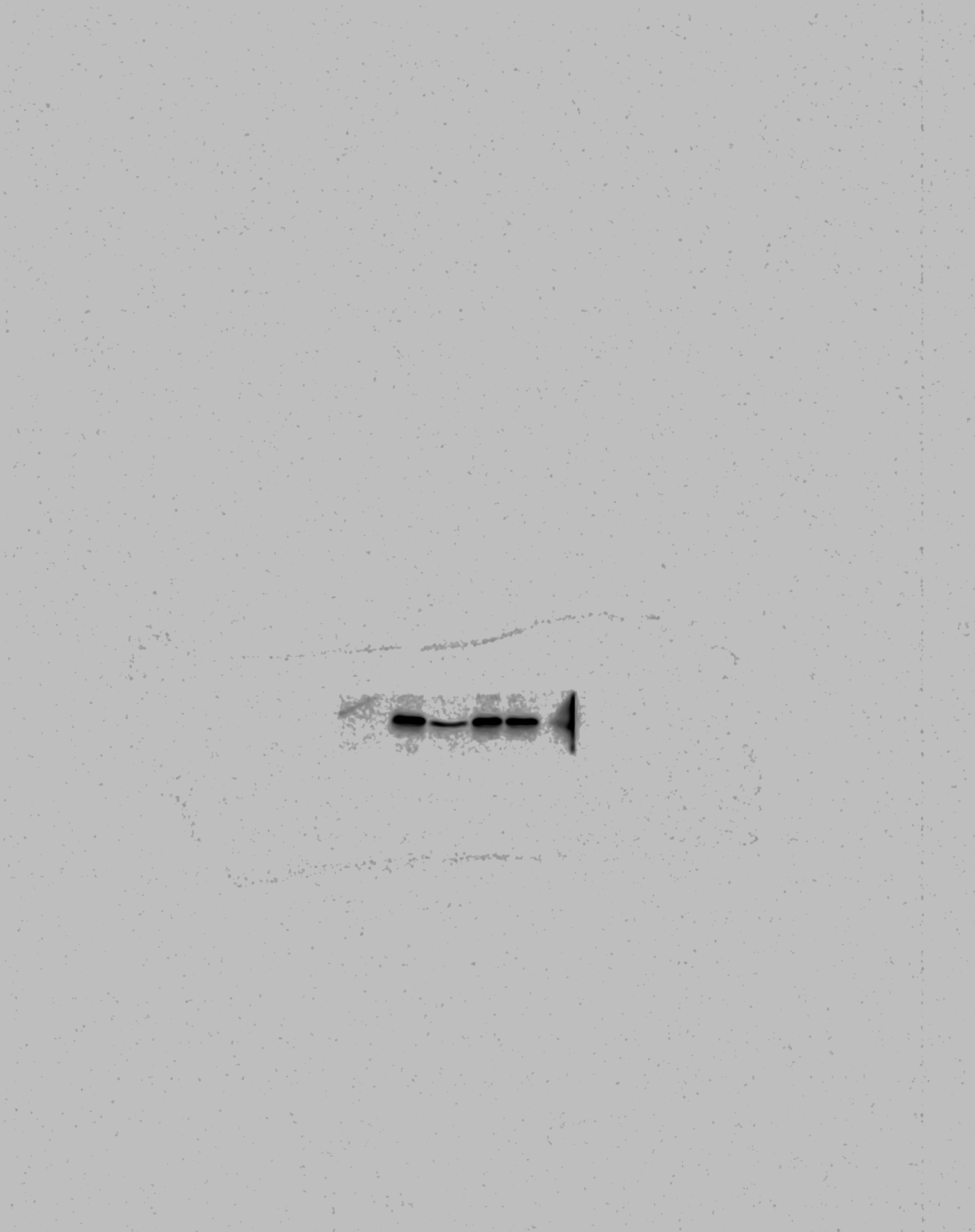

Supplement: Supplementary file 3 [file DataSheet_1.zip › figure3-ZIP1-2.tif]

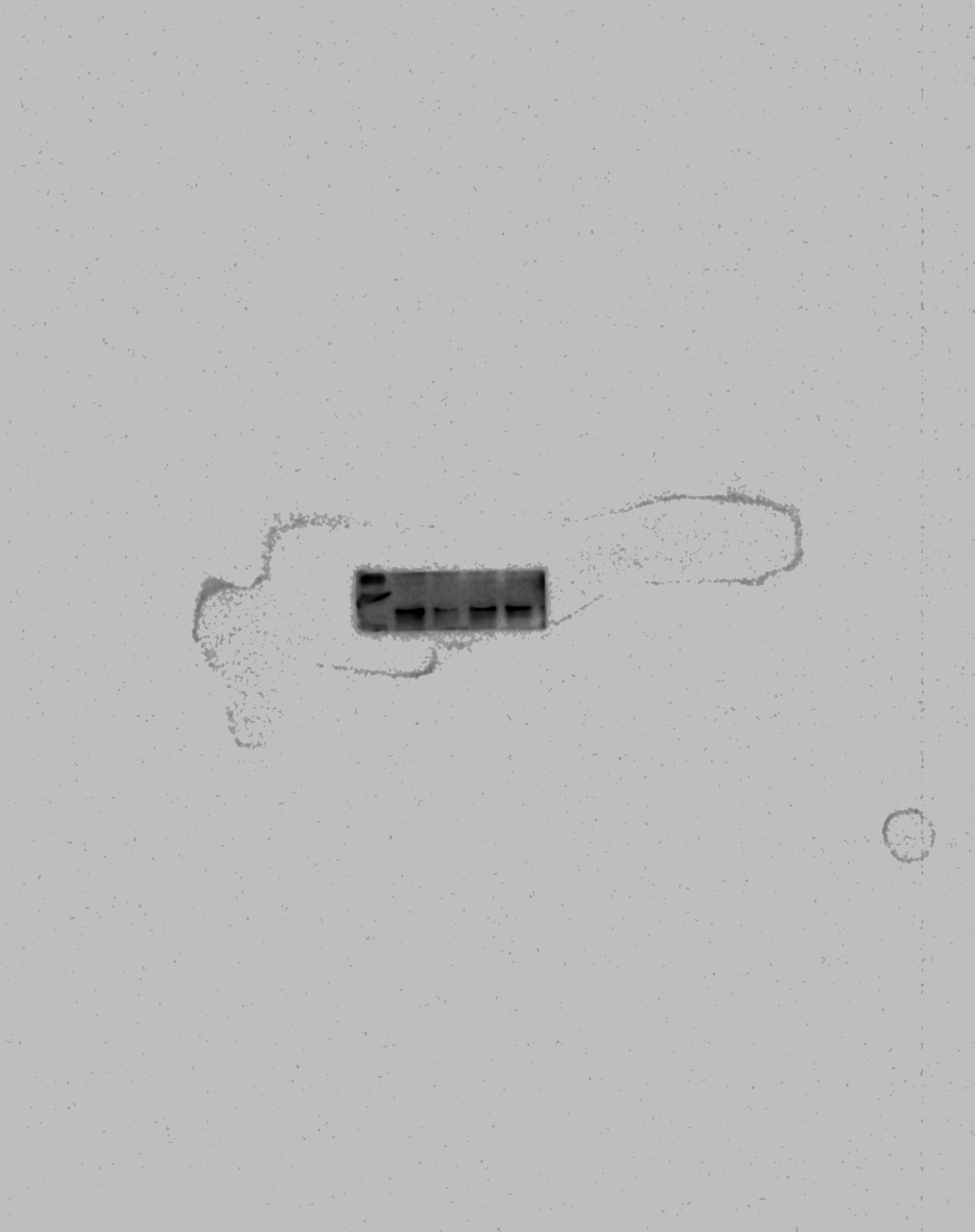

Supplement: Supplementary file 3 [file DataSheet_1.zip › figure3-ZIP1-3.tif]

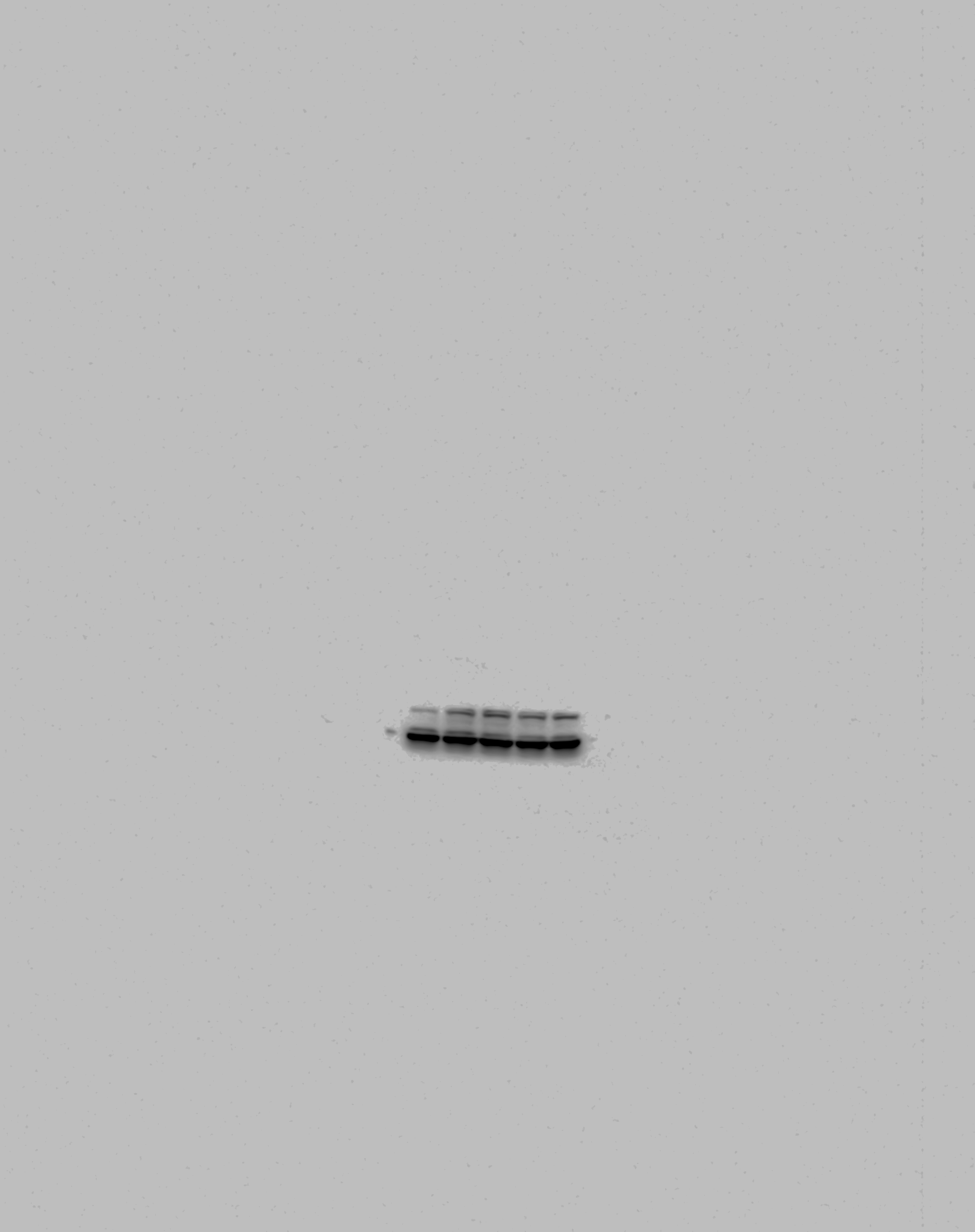

Supplement: Supplementary file 3 [file DataSheet_1.zip › figure4-actin-1.tif]

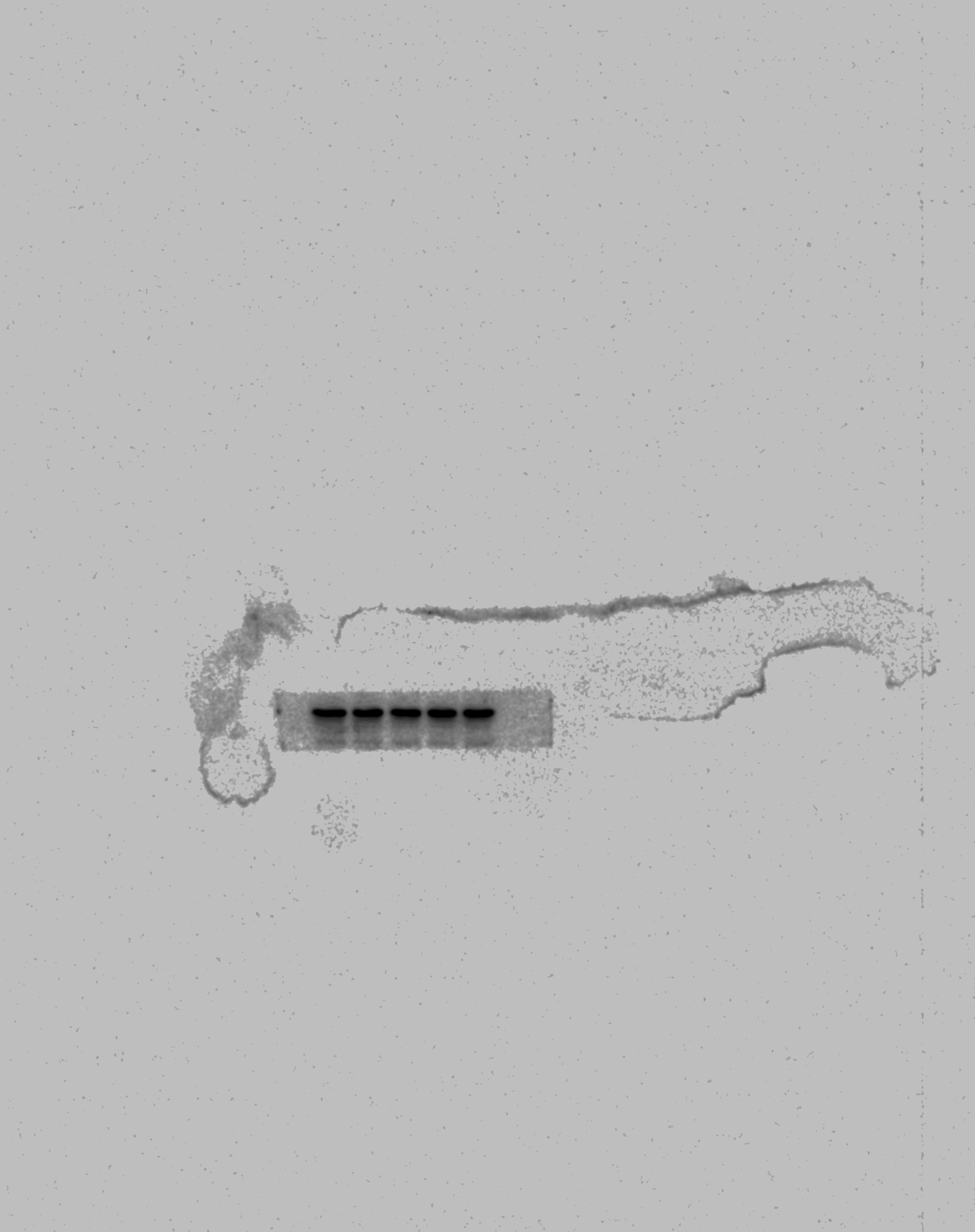

Supplement: Supplementary file 3 [file DataSheet_1.zip › figure4-actin-2.tif]

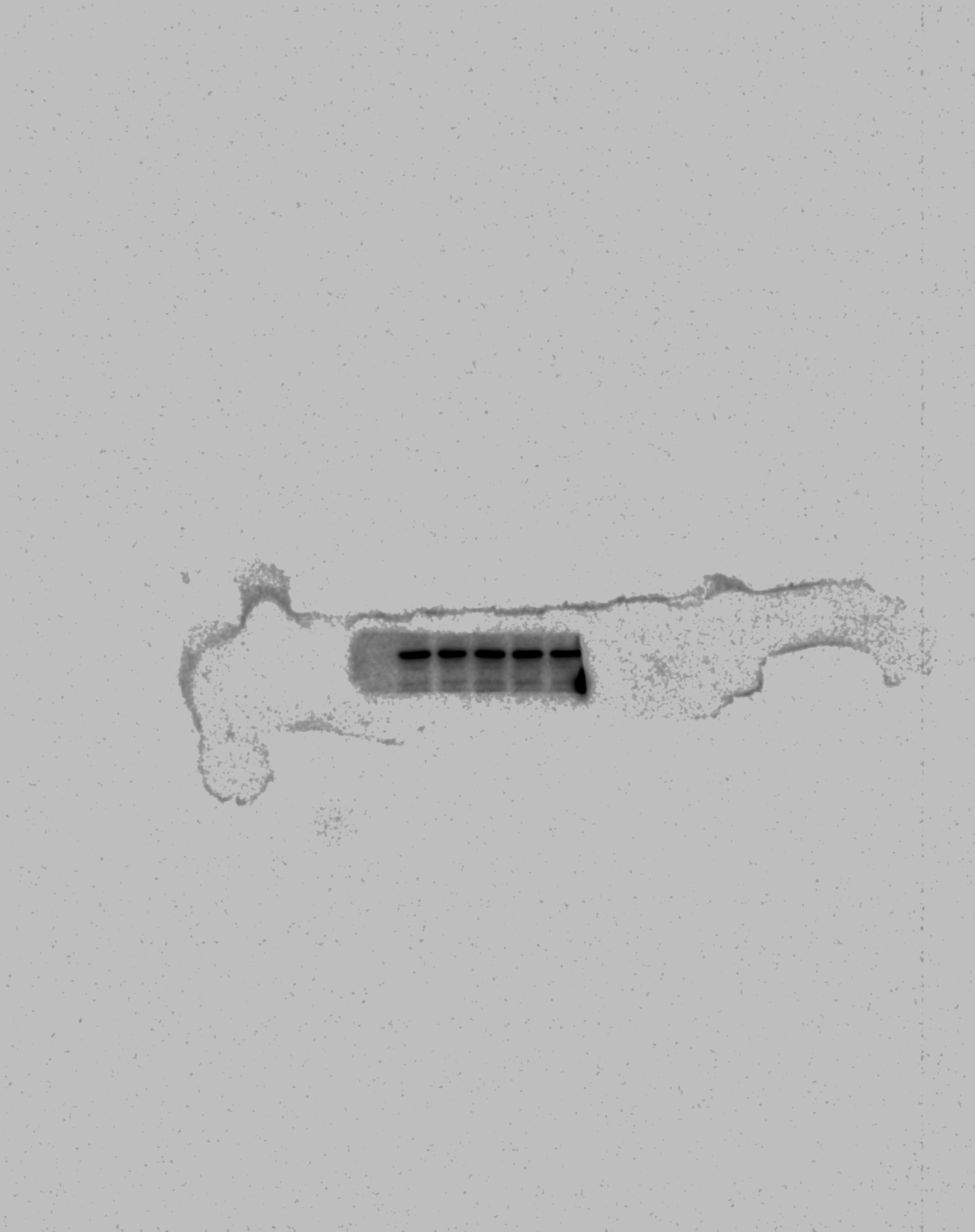

Supplement: Supplementary file 3 [file DataSheet_1.zip › figure4-actin-3.tif]

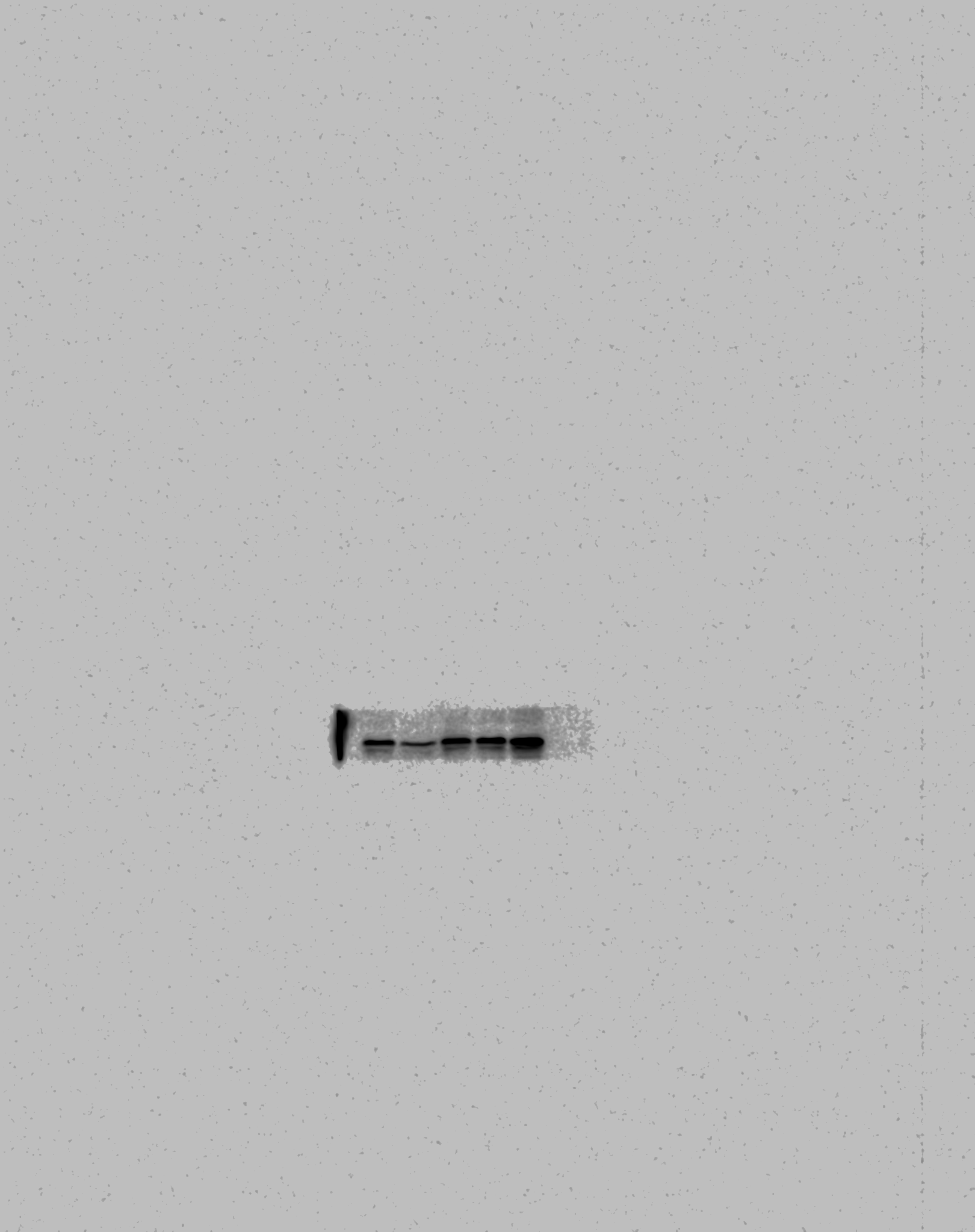

Supplement: Supplementary file 3 [file DataSheet_1.zip › figure4-ZIP1-1.tif]

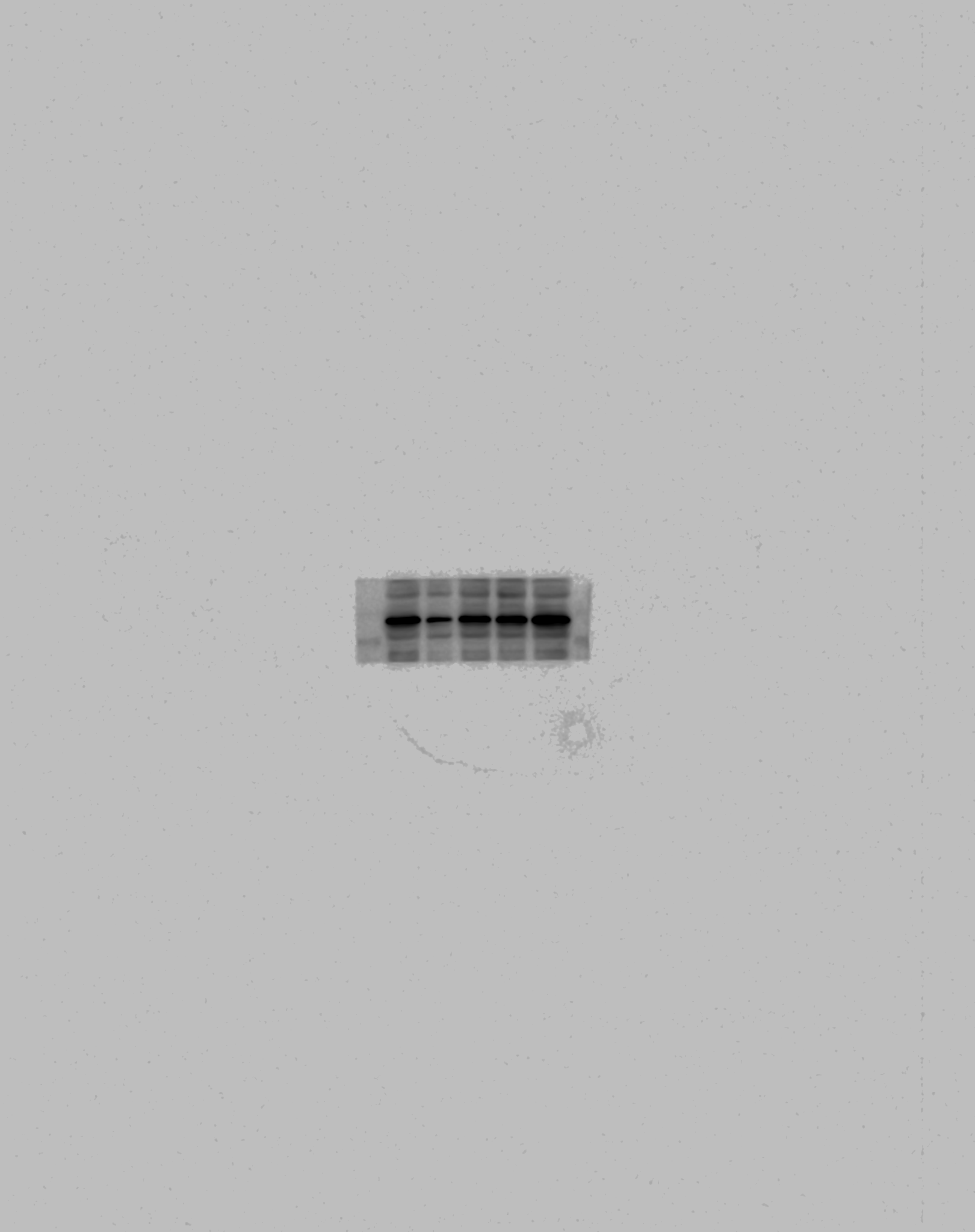

Supplement: Supplementary file 3 [file DataSheet_1.zip › figure4-ZIP1-2.tif]

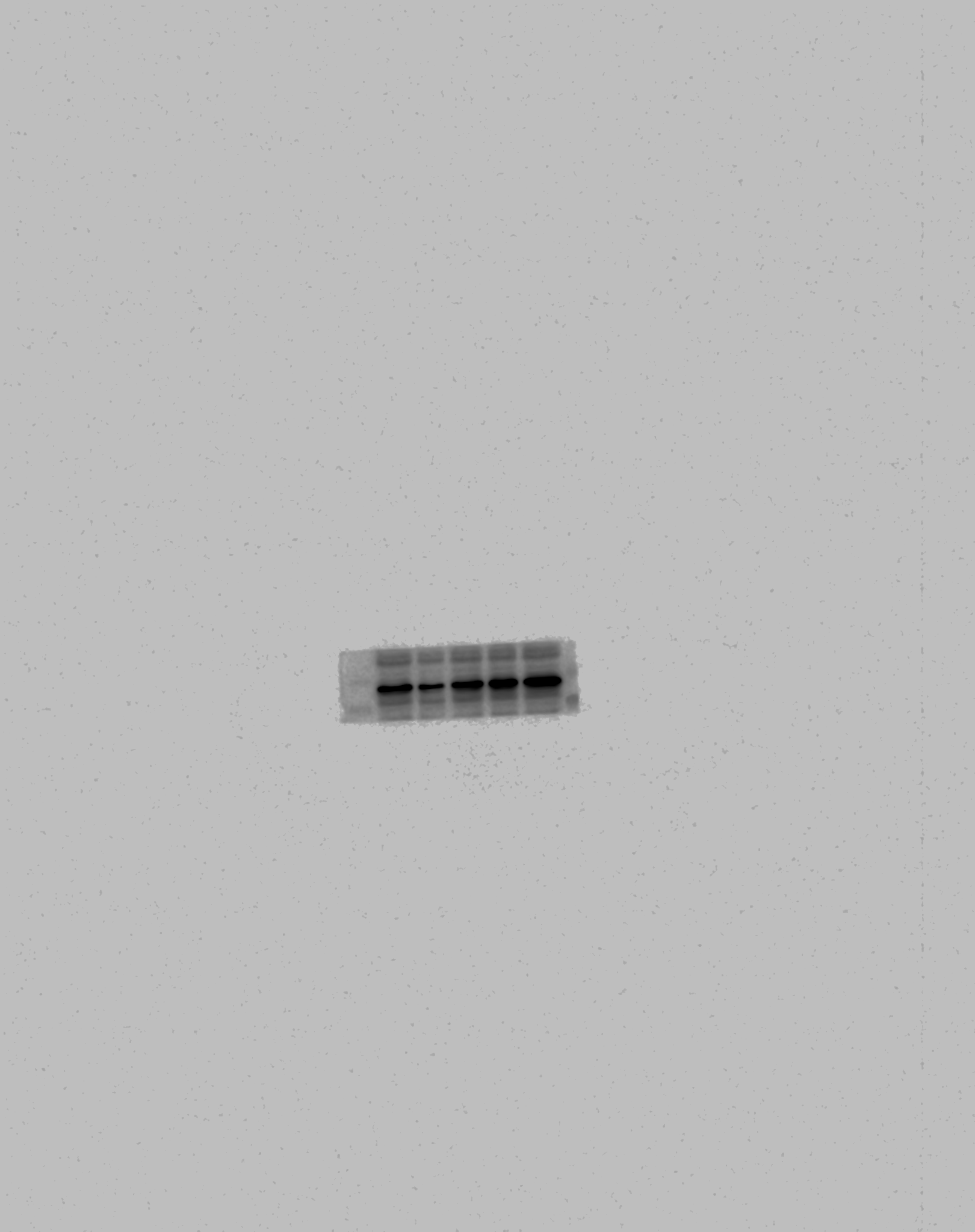

Supplement: Supplementary file 3 [file DataSheet_1.zip › figure4-ZIP1-3.tif]
