# Supplementary figures and images for: Corrigendum: Protective Role of Melatonin Against Postmenopausal Bone Loss via Enhancing Citrate Secretion in Osteoblasts
Source: Front Pharmacol. 2021 Mar 11;12:652249. doi: 10.3389/fphar.2021.652249 (PMC8025315; doi:10.3389/fphar.2021.652249)

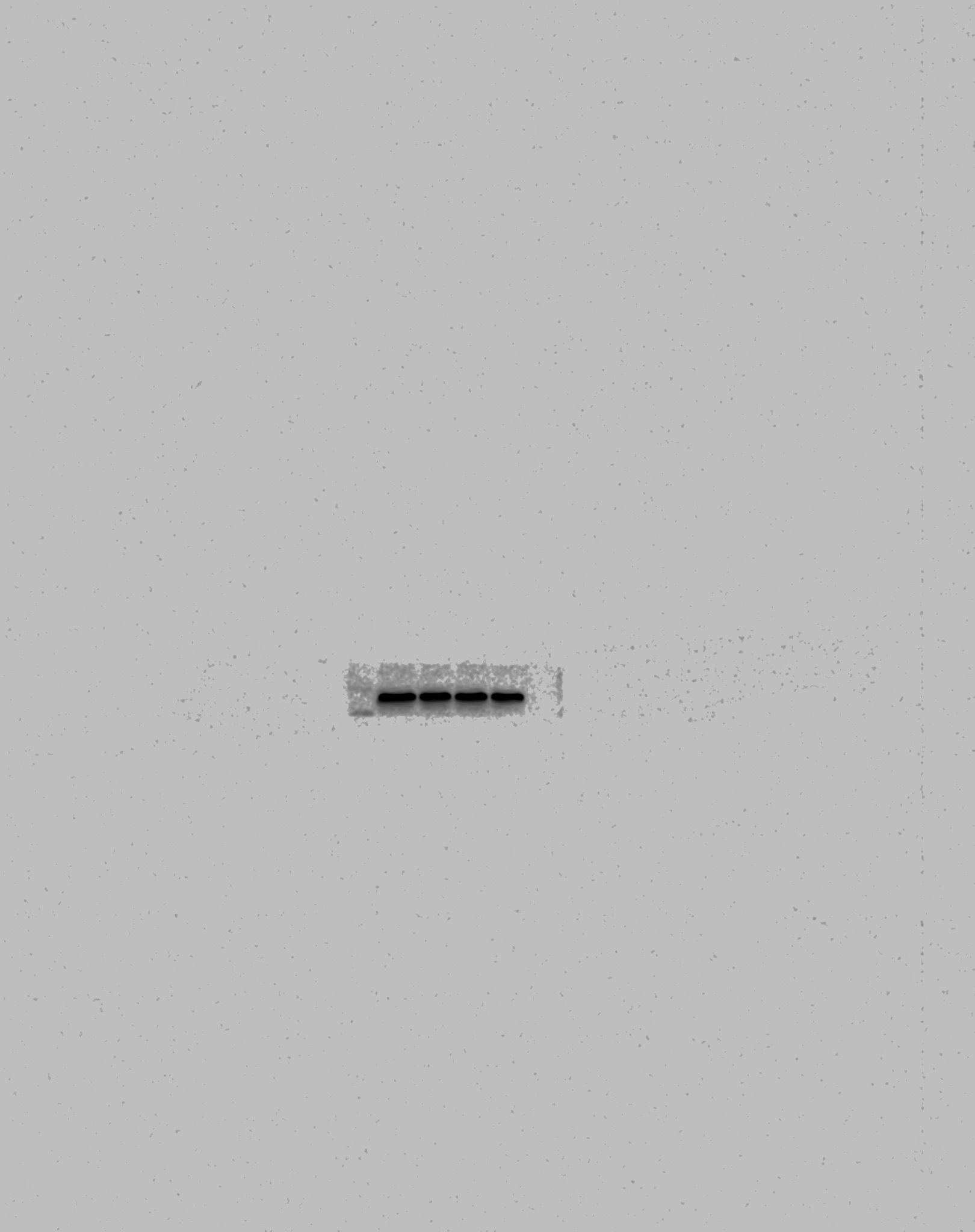

Supplement: Supplementary file 1 [file datasheet1.zip › figure3-actin-1.tif]

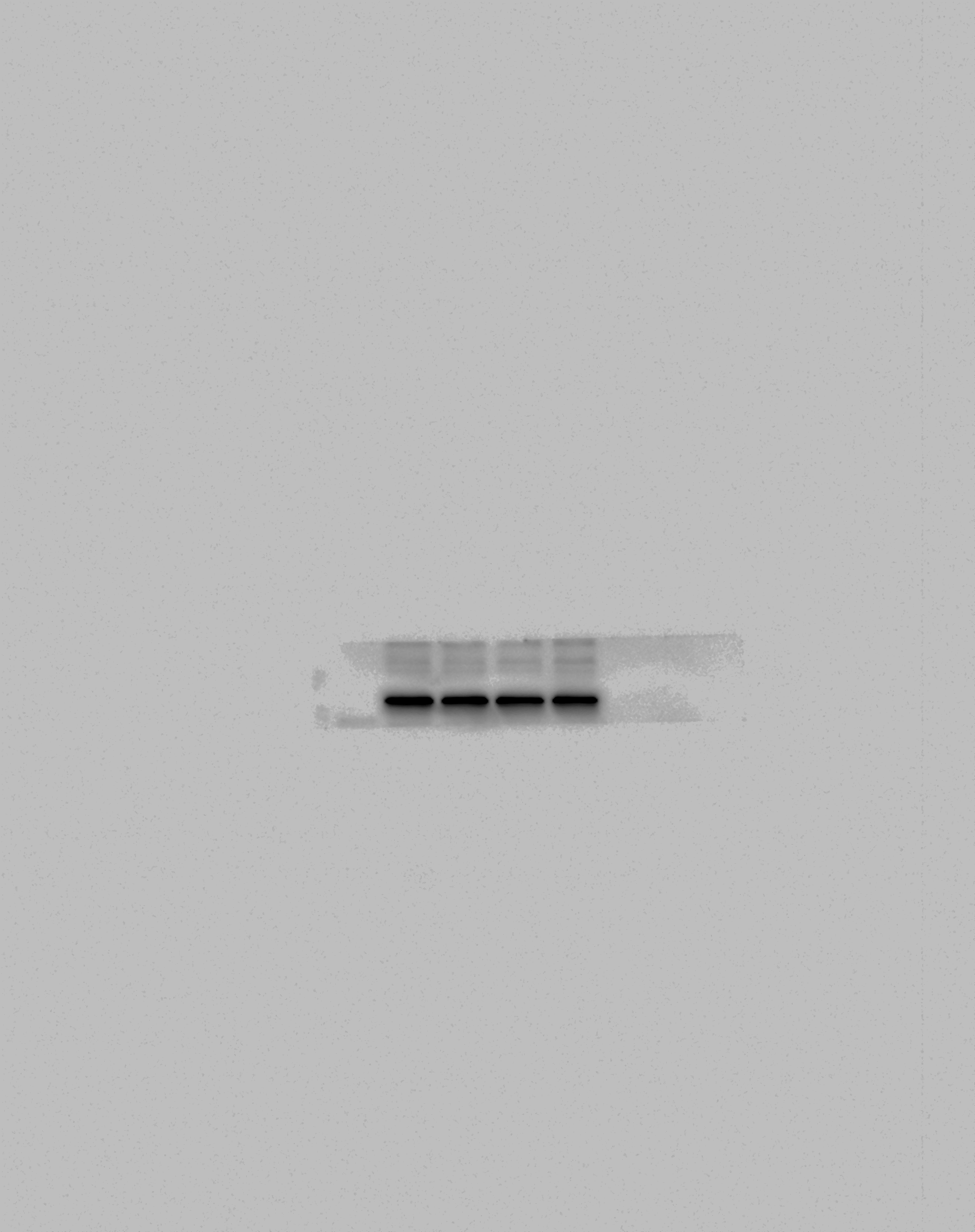

Supplement: Supplementary file 1 [file datasheet1.zip › figure3-actin-2.tif]

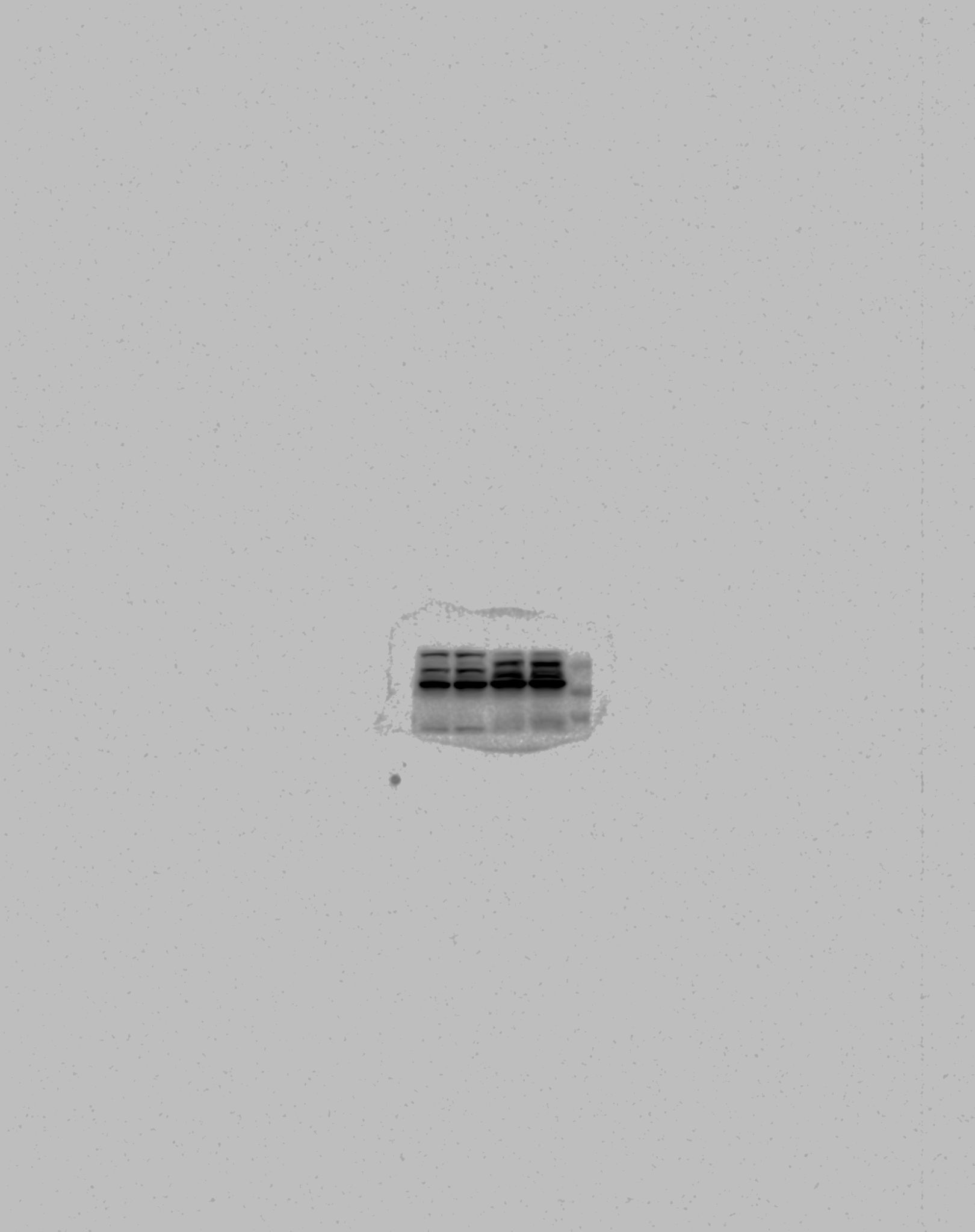

Supplement: Supplementary file 1 [file datasheet1.zip › figure3-actin-3.tif]

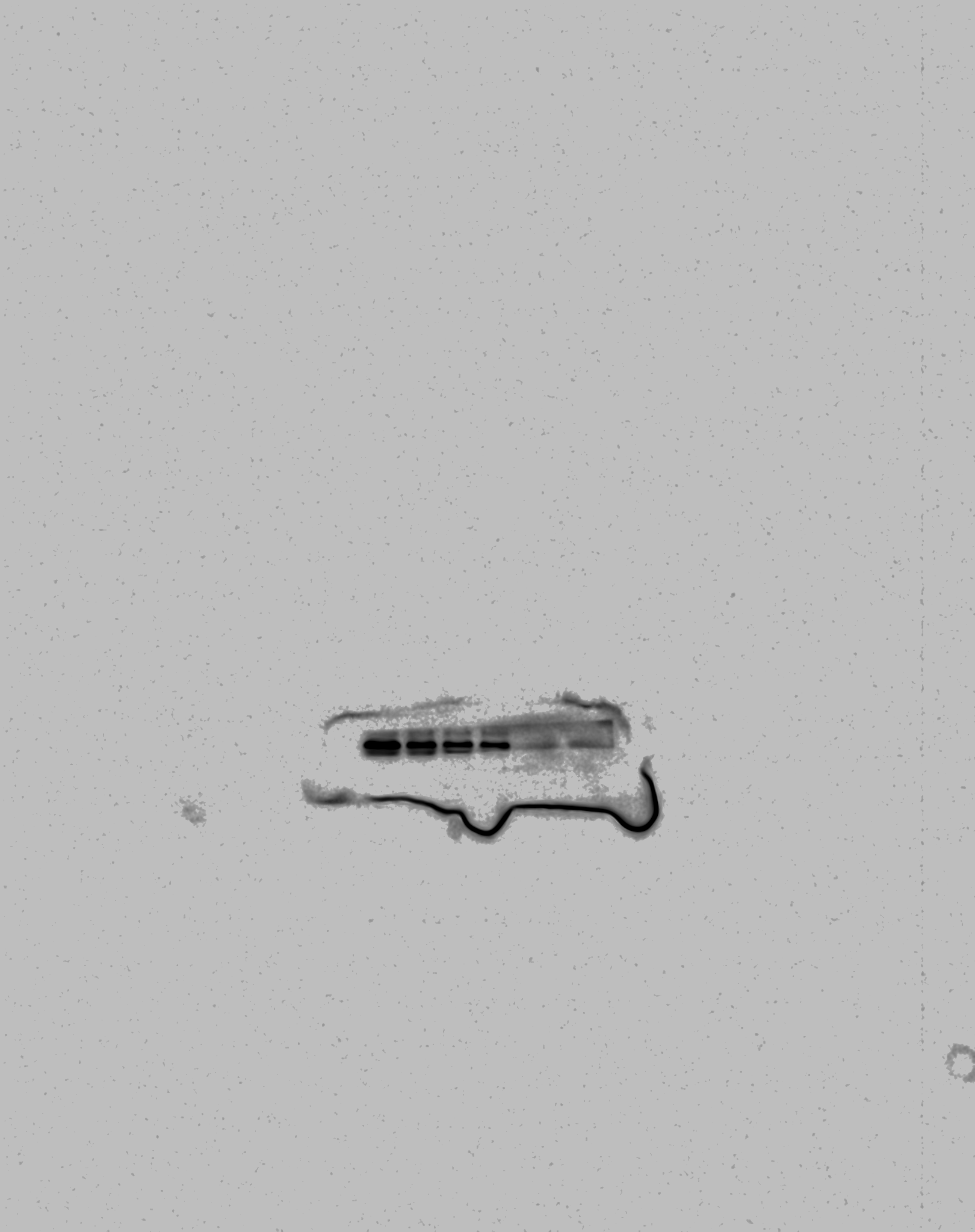

Supplement: Supplementary file 1 [file datasheet1.zip › figure3-CTP-1.tif]

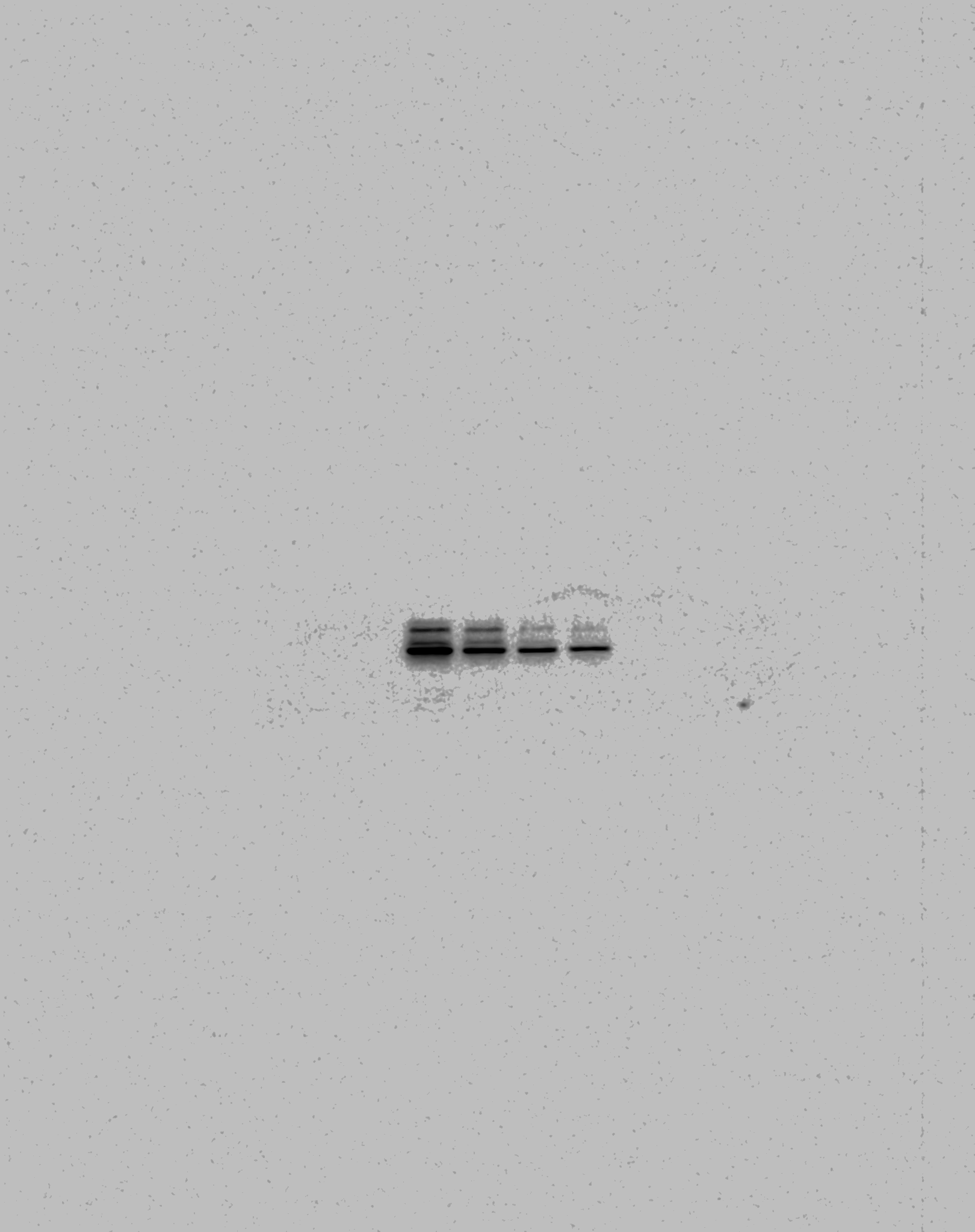

Supplement: Supplementary file 1 [file datasheet1.zip › figure3-CTP-2.tif]

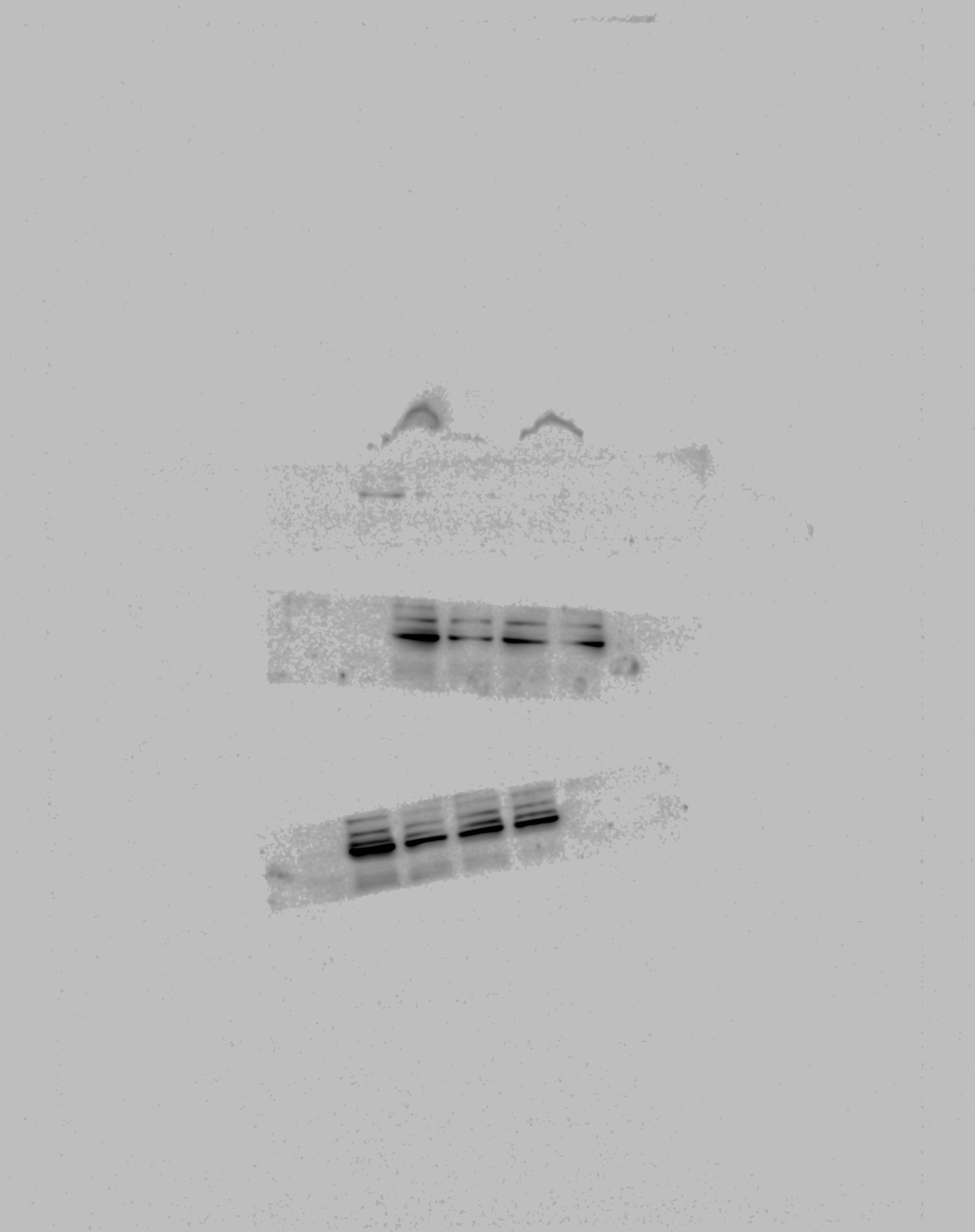

Supplement: Supplementary file 1 [file datasheet1.zip › figure3-CTP-3.tif]

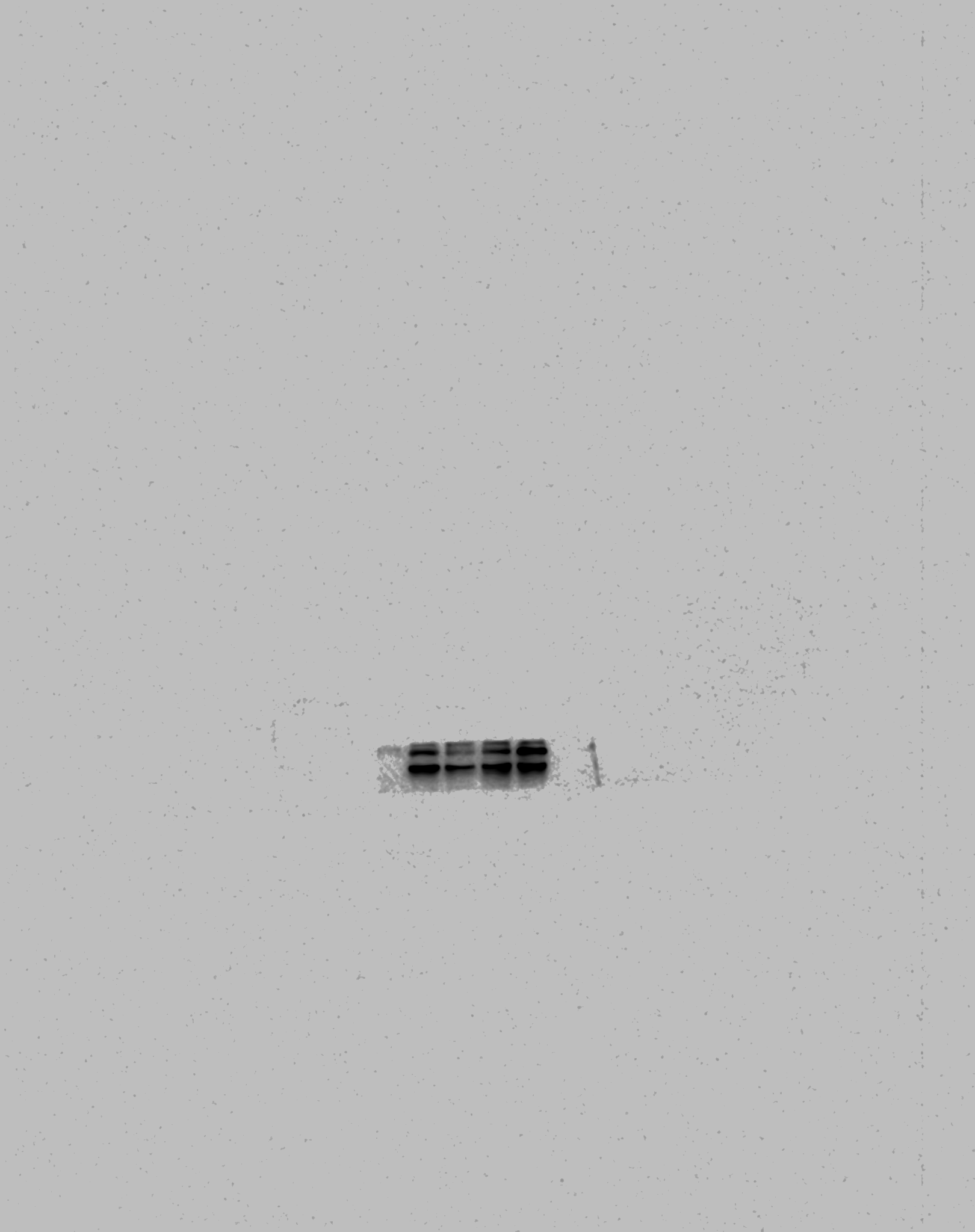

Supplement: Supplementary file 1 [file datasheet1.zip › figure3-ZIP1-1.tif]

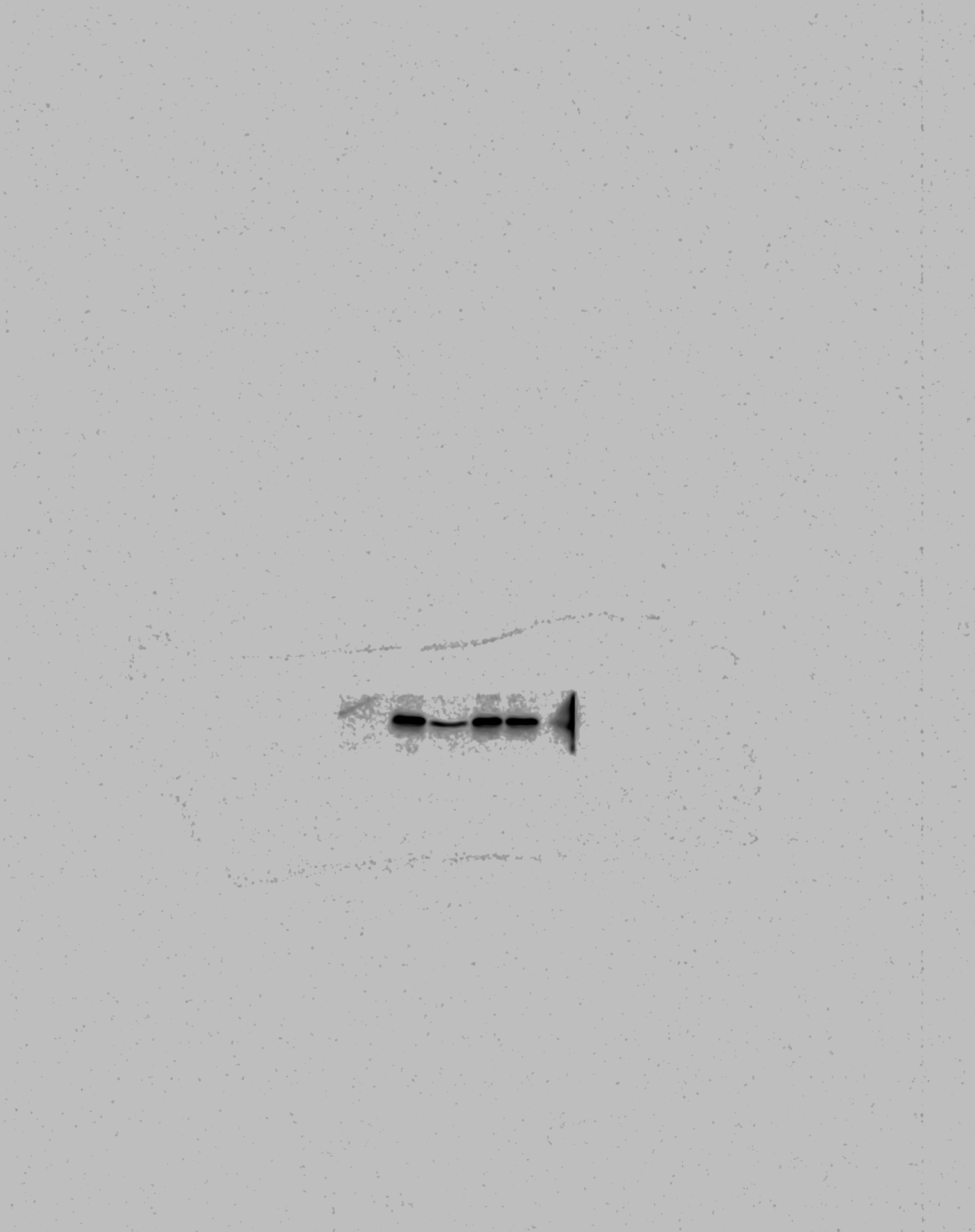

Supplement: Supplementary file 1 [file datasheet1.zip › figure3-ZIP1-2.tif]

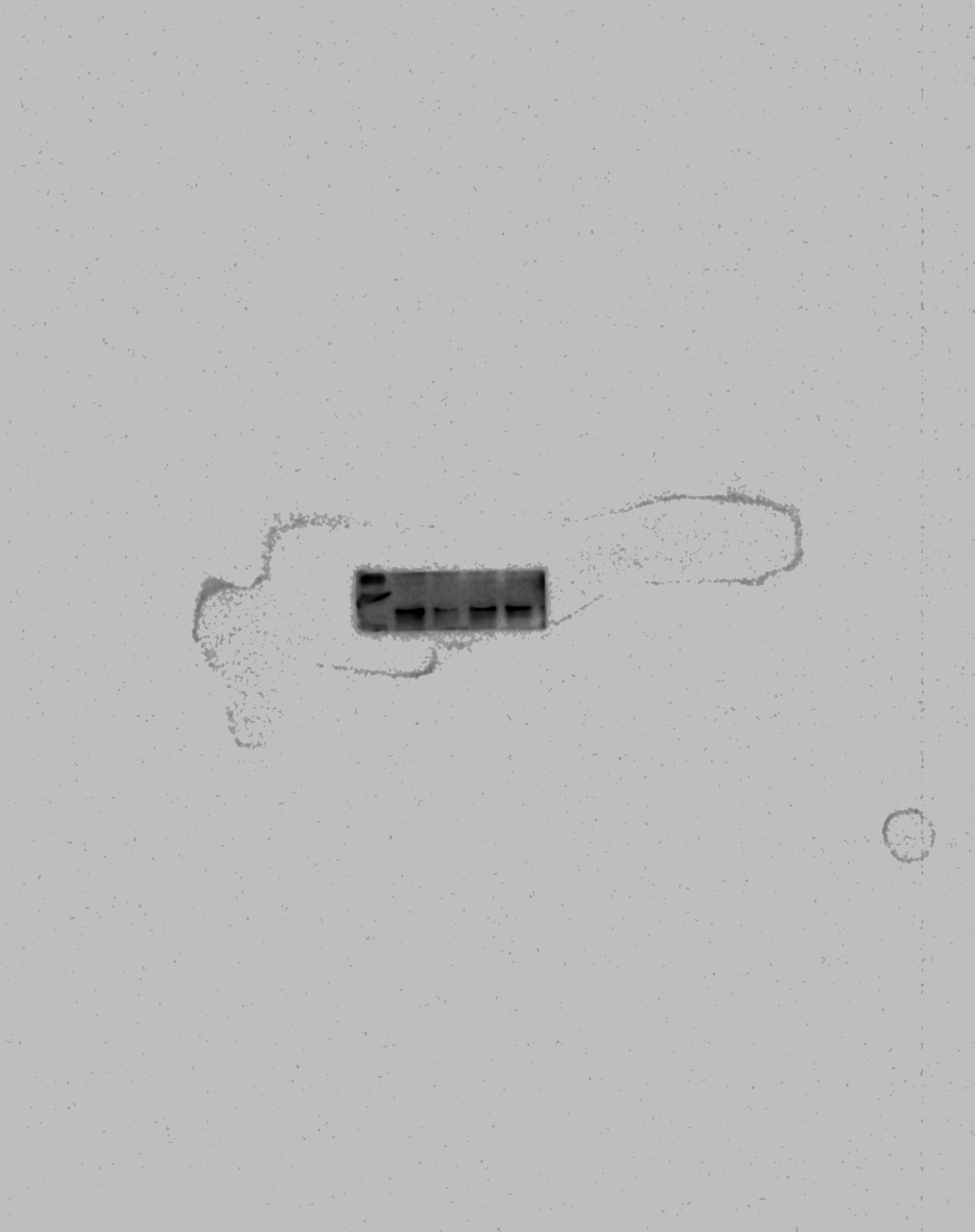

Supplement: Supplementary file 1 [file datasheet1.zip › figure3-ZIP1-3.tif]

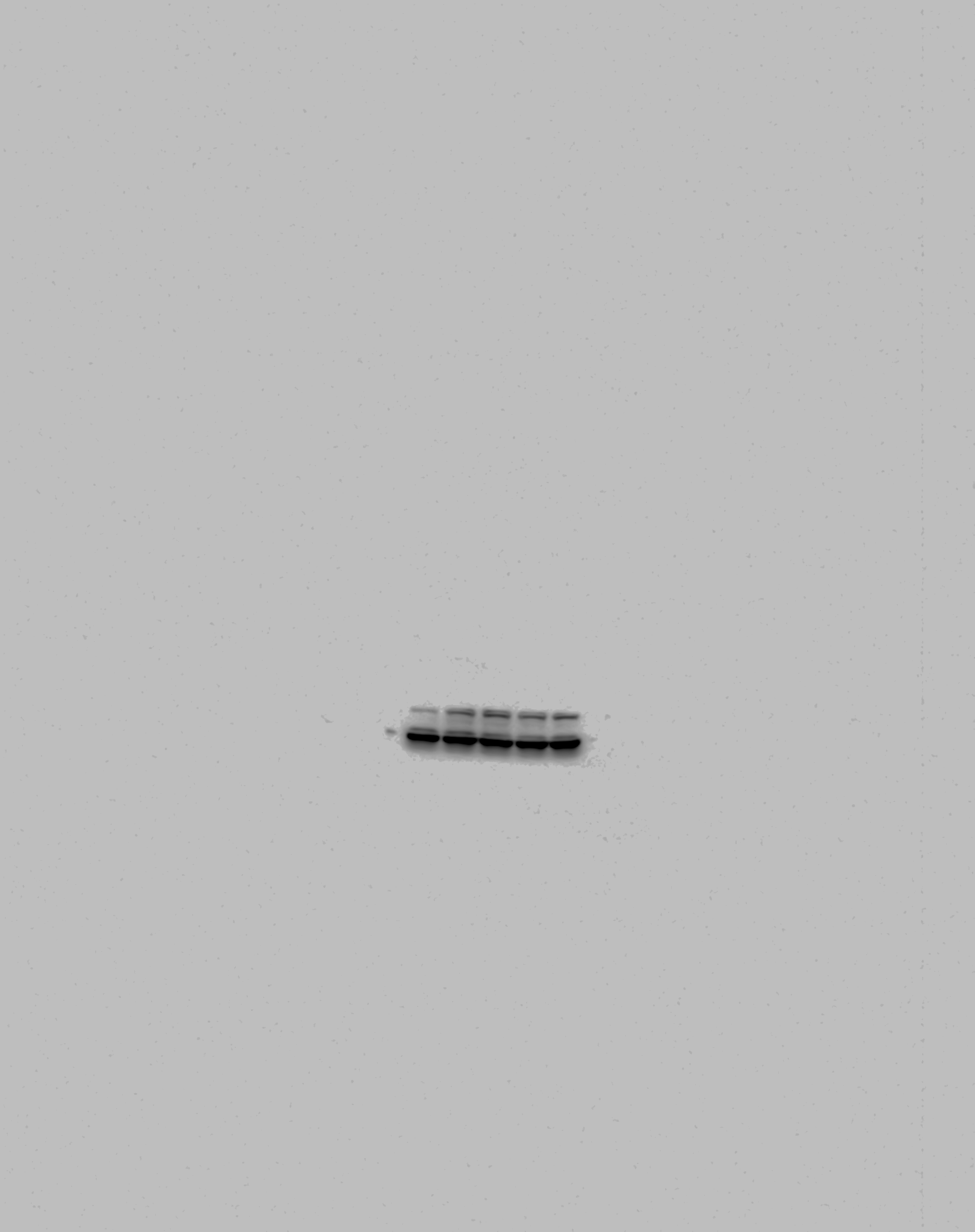

Supplement: Supplementary file 1 [file datasheet1.zip › figure4-actin-1.tif]

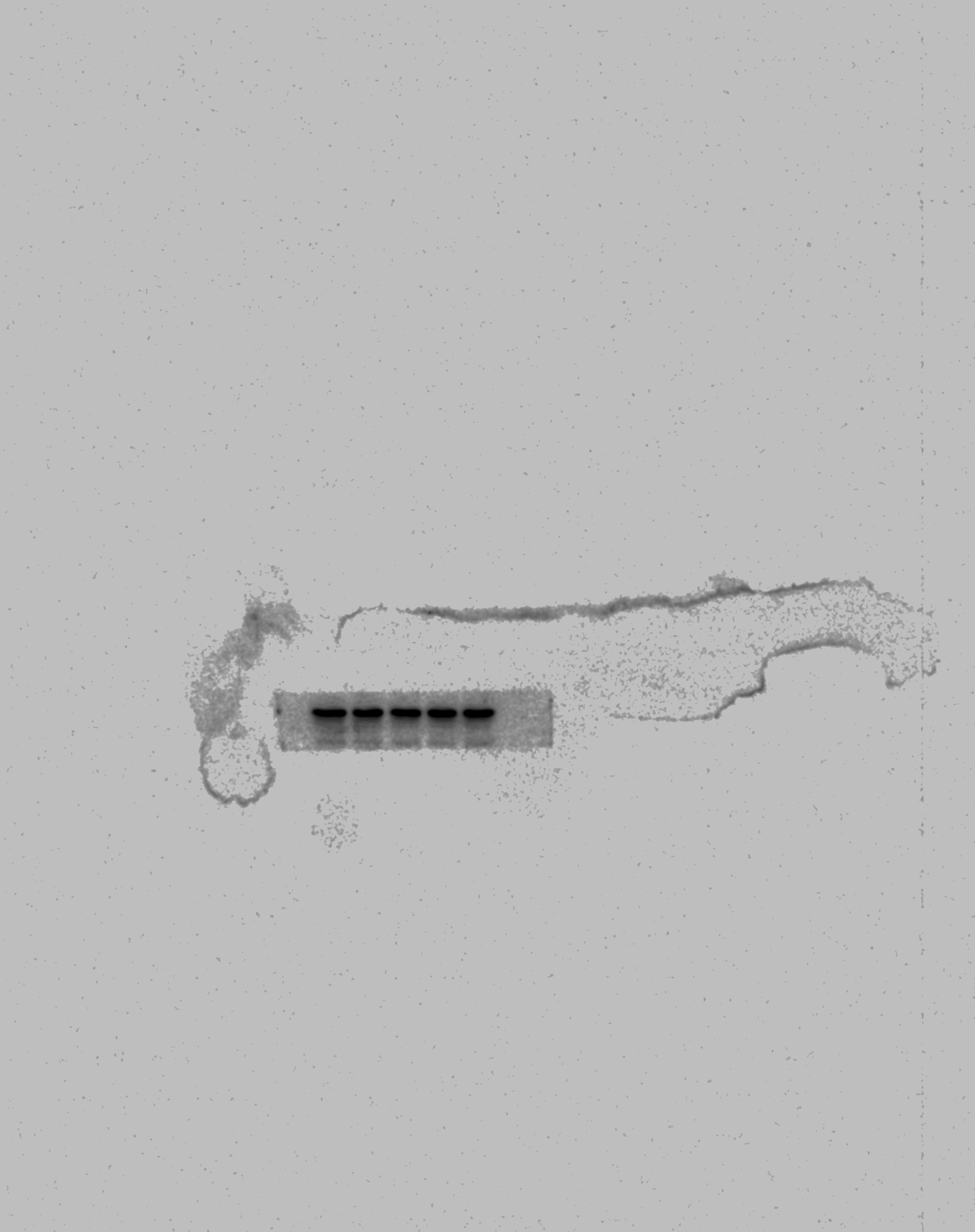

Supplement: Supplementary file 1 [file datasheet1.zip › figure4-actin-2.tif]

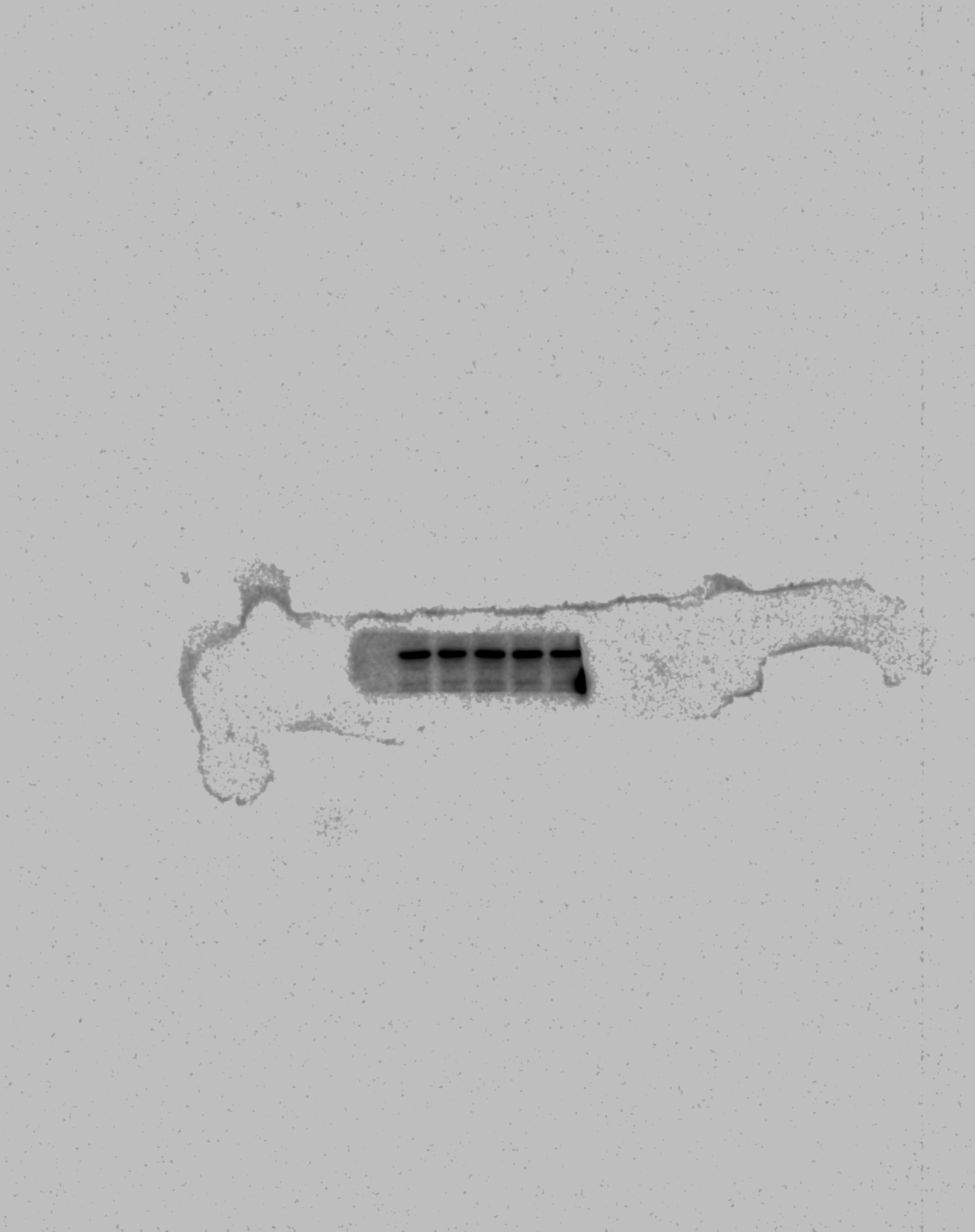

Supplement: Supplementary file 1 [file datasheet1.zip › figure4-actin-3.tif]

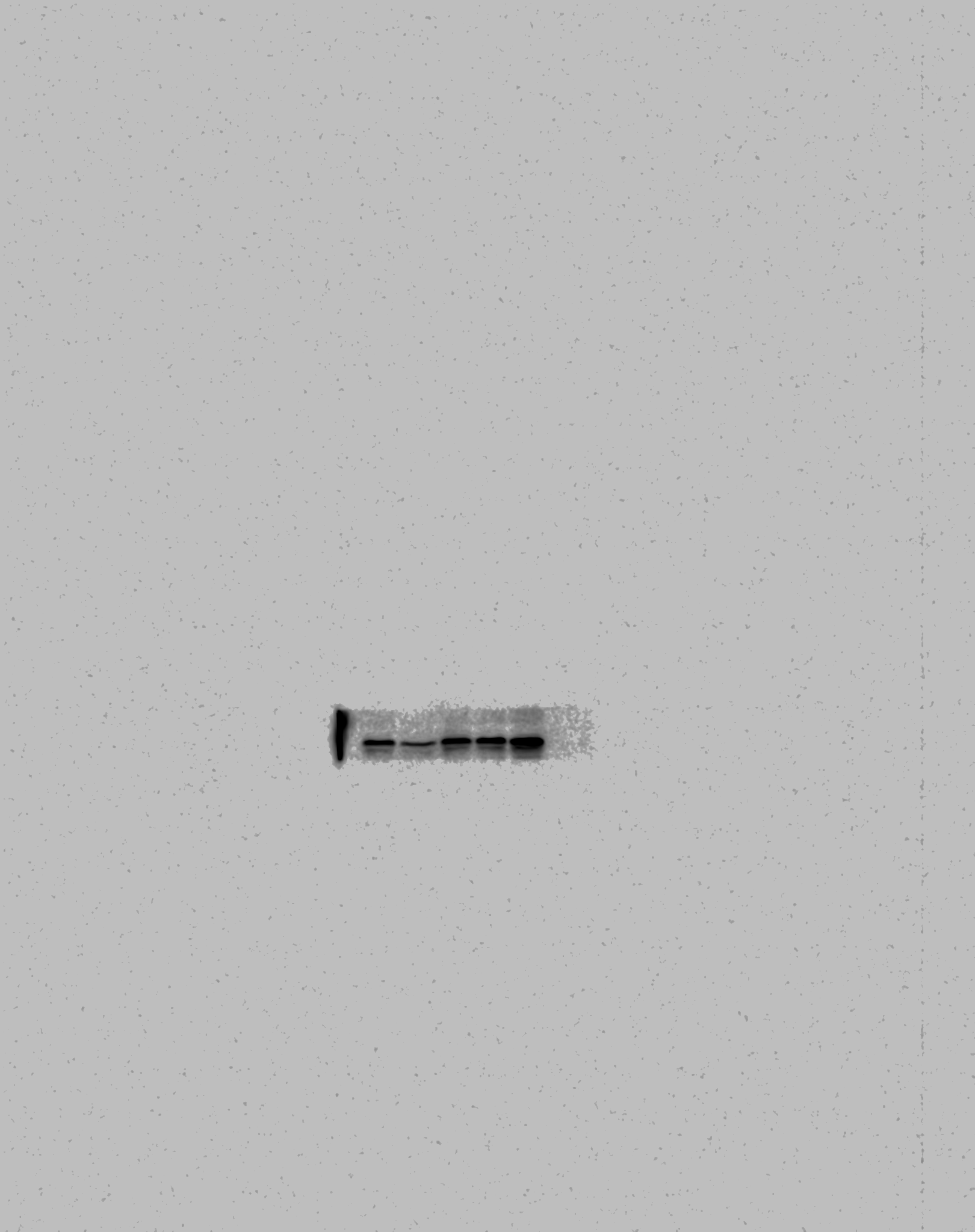

Supplement: Supplementary file 1 [file datasheet1.zip › figure4-ZIP1-1.tif]

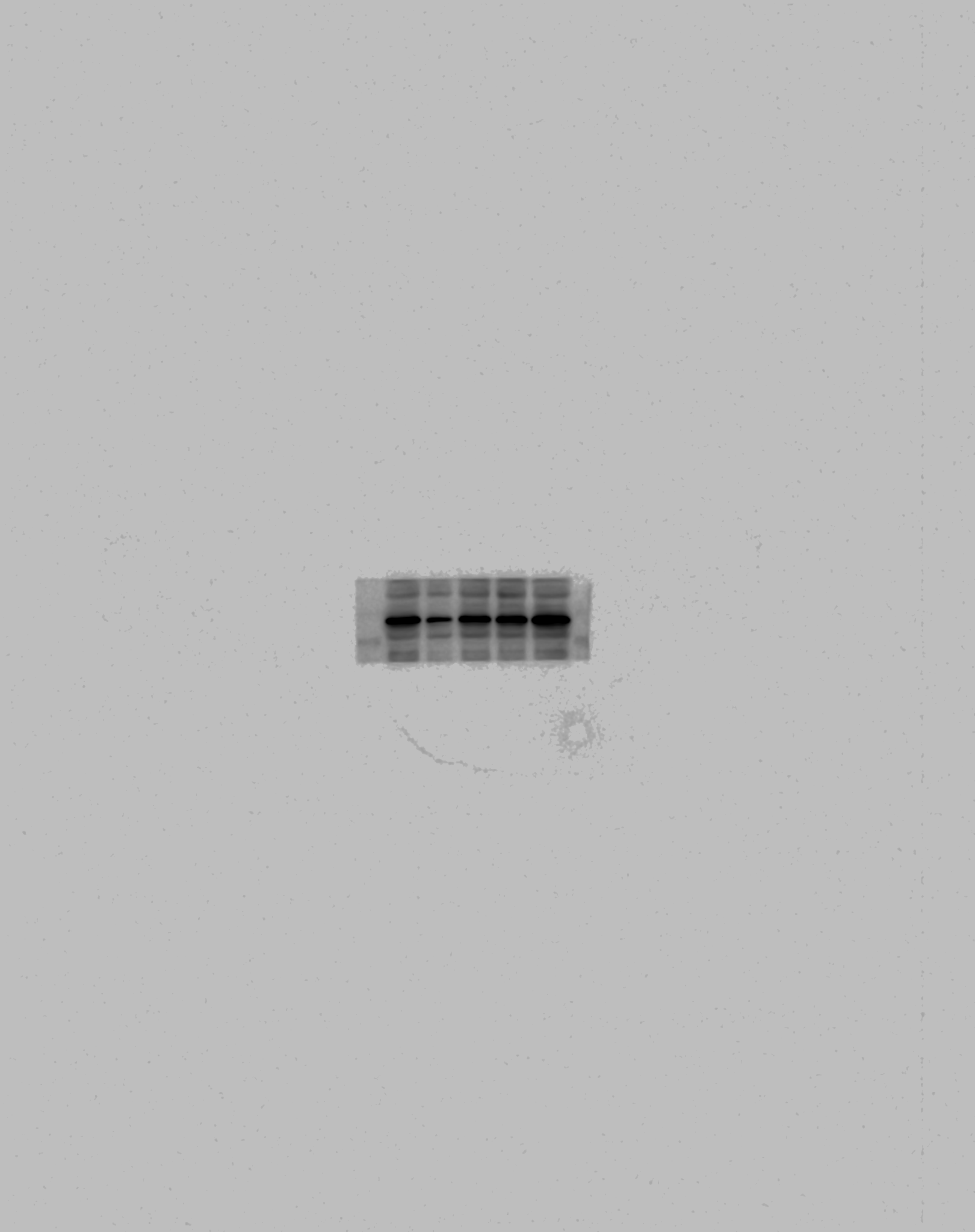

Supplement: Supplementary file 1 [file datasheet1.zip › figure4-ZIP1-2.tif]

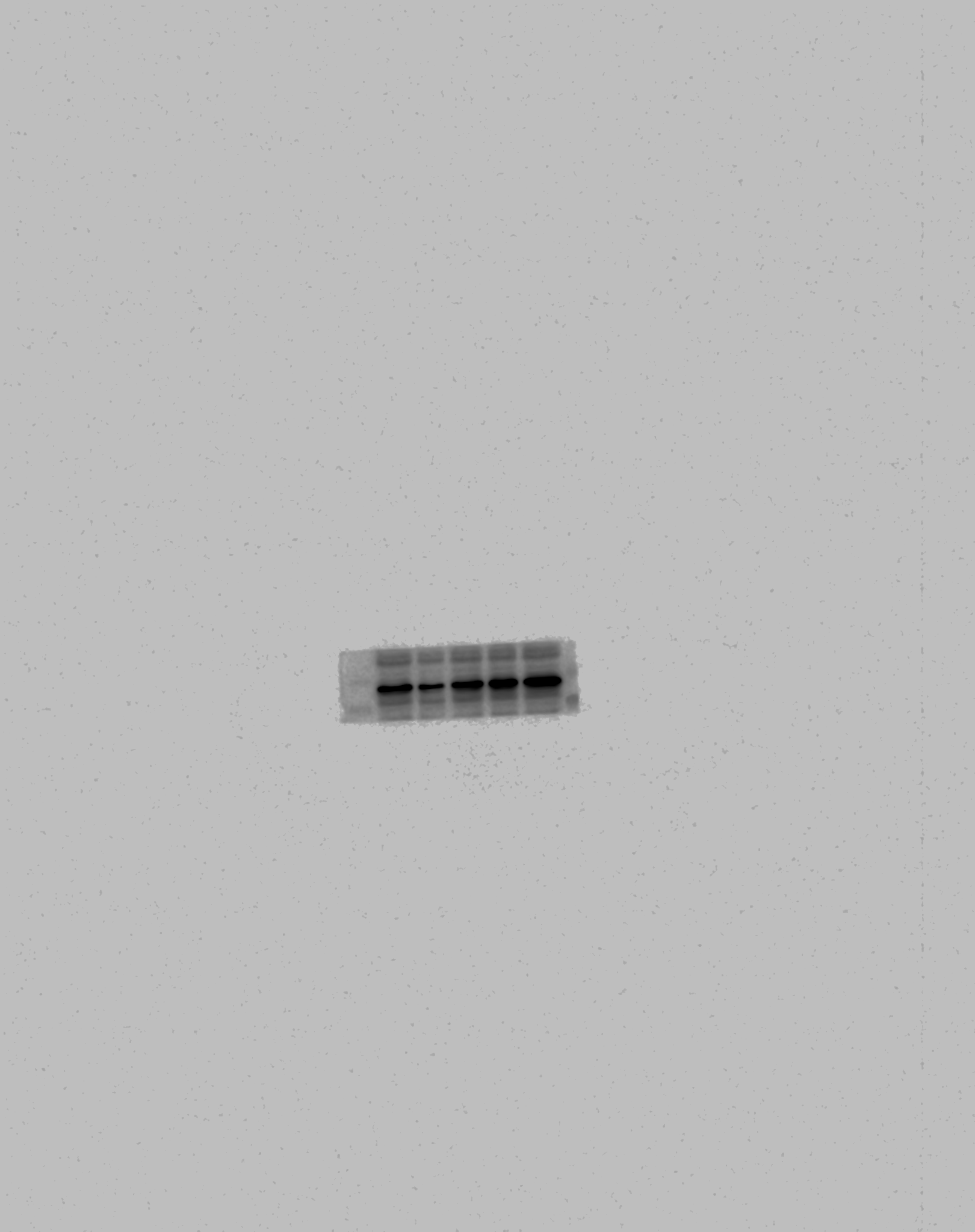

Supplement: Supplementary file 1 [file datasheet1.zip › figure4-ZIP1-3.tif]

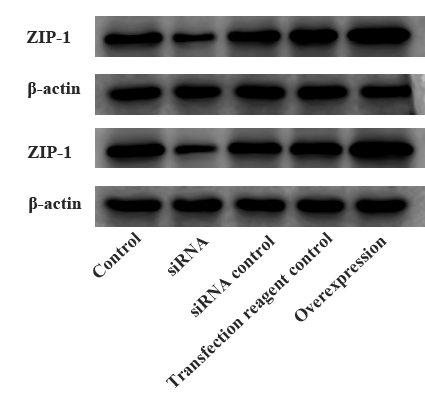

Supplement: Supplementary file 2 [file image2.tif]

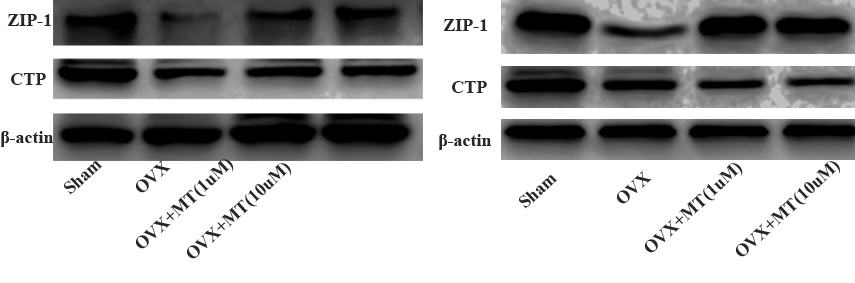

Supplement: Supplementary file 3 [file image1.tif]
